# High compositional and functional similarity in the microbiome of deep-sea sponges

**DOI:** 10.1093/ismejo/wrad030

**Published:** 2024-01-12

**Authors:** Cristina Díez-Vives, Ana Riesgo

**Affiliations:** Department of Systems Biology, Centro Nacional de Biotecnología, c/ Darwin, 3, 28049 Madrid, Spain; Department of Life Sciences, The Natural History Museum, London SW7 5BD, United Kingdom; Department of Life Sciences, The Natural History Museum, London SW7 5BD, United Kingdom; Department of Biodiversity and Evolutionary Biology, Museo Nacional de Ciencias Naturales (CSIC), c/José Gutiérrez Abascal 2, 28006 Madrid, Spain

**Keywords:** sponge microbiome, microbial gene expression, amplicon sequencing, host-microbiome systems, microbial ecology, deep-sea ecosystems

## Abstract

Sponges largely depend on their symbiotic microbes for their nutrition, health, and survival. This is especially true in high microbial abundance (HMA) sponges, where filtration is usually deprecated in favor of a larger association with prokaryotic symbionts. Sponge-microbiome association is substantially less understood for deep-sea sponges than for shallow water species. This is most unfortunate, since HMA sponges can form massive sponge grounds in the deep sea, where they dominate the ecosystems, driving their biogeochemical cycles. Here, we assess the microbial transcriptional profile of three different deep-sea HMA sponges in four locations of the Cantabrian Sea and compared them to shallow water HMA and LMA (low microbial abundance) sponge species. Our results reveal that the sponge microbiome has converged in a fundamental metabolic role for deep-sea sponges, independent of taxonomic relationships or geographic location, which is shared in broad terms with shallow HMA species. We also observed a large number of redundant microbial members performing the same functions, likely providing stability to the sponge inner ecosystem. A comparison between the community composition of our deep-sea sponges and another 39 species of HMA sponges from deep-sea and shallow habitats, belonging to the same taxonomic orders, suggested strong homogeneity in microbial composition (i.e. weak species-specificity) in deep sea species, which contrasts with that observed in shallow water counterparts. This convergence in microbiome composition and functionality underscores the adaptation to an extremely restrictive environment with the aim of exploiting the available resources.

## Introduction

Sponges are well known for their intimate associations with a diverse range of microbial symbionts, which were probably established at the advent of the phylum Porifera, more than 580 million years ago [1-3]. While sponges represent a substantial part of the benthic fauna in both marine and freshwater habitats [4, 5], part of their ecological success is due to their, mostly, unbreakable partnership with these symbiotic microbes [4, 6, 7]. Sponge prokaryotic symbionts serve as energy sources for their host and contribute appreciably to their metabolism and biochemical repertoire, influencing their survival and role in biochemical fluxes [7-10]. As a result, sponges are essential for the ocean’s health due to their function in both habitat formation and biogeochemical cycling in benthic ecosystems [4, 7, 11]. In deep-sea habitats particularly, sponges can form massive associations, called “sponge grounds,” that parallels coral reefs in terms of biodiversity and ecosystem services [9].

Diversity and function of sponge-associated symbionts is increasingly understood for shallow sponges, but we know substantially less about the composition and function of the sponge microbiomes in the deep-sea, due to the inherent difficulties in sample collection. The microbial communities of shallow water sponges display well-known philopatry, with species-specific patterns [1, 12-15], more marked in high microbial abundance (HMA) sponges than low microbial abundance (LMA) sponges [16], and even genotype-specific associations within sponge populations [17-19]. At the functional level, several metatranscriptomic studies conducted on shallow sponge microbiomes [20-26] have greatly increased our understanding of marine sponge symbiosis and functional activities. For deep-sea sponges, however, most of our knowledge comes from amplicon sequencing (16S rRNA gene profiling) and metagenomic analyses that can only show the diversity and functional potential of sponge symbionts [27-31], but also it is yet to be established what prokaryotic genes are genuinely being utilized. We know that in *Geodia barretti,* from the Norwegian west coast [32], the most abundant mRNAs encoded key metabolic enzymes of nitrification from ammonia-oxidizing archaea, confirming the high rates of nitrification observed in this sponge [33]. The metatranscriptomes of *Geodia parva*, from the sponge grounds of Central Arctic seamounts [34], showed high expression levels for refractory dissolved organic carbon (DOM) utilization, ammonia and nitrate assimilation, as well as sulfur and carbon fixation pathways. *Hymedesmia (Stylopus) methanophila* and *Iophon methanophila* harbored two closely related methane-oxidizing (MOX) bacteria, with high methane assimilation expression rates in deep-sea asphalt seeps knolls in the southern Gulf of Mexico [35].

Because the current knowledge in deep-sea sponges generally delves on specific functions (mostly the carbon and nitrogen cycle) or focuses on a single sponge species, the analysis of common patterns and trends in microbiome specificity and function is virtually impossible. Here, we aimed to determine the composition and metabolic transcriptional profile of the symbiotic microbiome of three different abundant HMA sponge species from the deep North Atlantic Ocean (*G. barretti*, *Geodia pachydermata*, and *Pachastrella ovisternata*), comparing it to that of shallow water HMA and LMA sponges (*Plakortis angulospiculatus* and *Halisarca caerulea*), to assess functional differences as well as evolutionary convergences. In addition, we aim to understand whether general trends observed in shallow water species such as species-specificity and influence of geography on their microbiome [6, 7] also occur in deep-sea sponges. To this end, we tested these effects on the microbiome composition and functionality of the deep-sea sponges across different locations in the Cantabrian Sea. The composition and properties of these deep-sea microbiomes were further compared against another 21 species of HMA sponges from deep-sea and 18 HMA species from shallow waters belonging to the same taxonomic orders, to assess taxonomical and environmental constraints in their microbiomes.

## Materials and methods

### Sample collection

**Figure 1:**
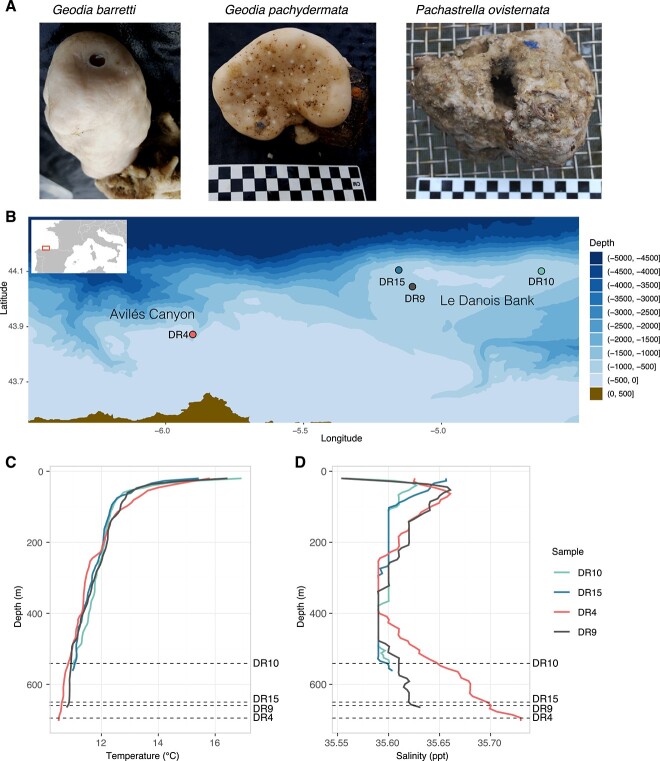
(A) Pictures of the sponge species collected during the SponGES0617 campaign and used in this study, and (B) map of the Cantabrian Sea covering the sponge collection sites; temperature (C) and salinity (D) profiles for the four sites where sponge samples were taken.

Sponge samples of *G. barretti* Bowerbank, 1858, *G. pachydermata* Sollas, 1886, and *P. ovisternata* Lendenfeld, 1894 (Fig. 1A) were collected on the SponGES0617 campaign in the Cantabrian Sea on board of the Ángeles Alvariño research vessel (June 12–27th, 2017), covering both the Avilés Canyon system (DR4) and Le Danois Bank (DR9, DR10, and DR15; Fig. 1B, Table S1). Samples were collected with a rock dredge during 5 minutes of trawling at bottom and brought to surface for sorting. Sponges were immediately placed in individual buckets with fresh seawater to avoid contamination and ensure DNA and RNA viability. Samples for genomics and transcriptomics were rapidly placed in RNAlater (Ambion) and 96% ethanol (replaced three times) and further stored at −20°C until processing. The sampling sites are Vulnerable Marine Ecosystems that harbor a great diversity of benthic organisms [36, 37]. The sampling sites are located between 500- and 700-m depth, in an area where one main water mass is reported: The Eastern North Atlantic Central Water (ENACW), extending from the base of the winter mixed layer (~200 m) to the sea floor. While the temperature is very similar across sites, sponges at El Corbiro Canyon (DR4) are subjected to slightly higher saline waters (Fig. 1C and D, also see [29]). We selected *G. barretti*, *G. pachydermata*, and *P. ovisternata*, for an in-depth analysis because they all belong to the order *Tetractinellida*, both *Geodia* spp. within the family *Geodiidae* and *P. ovisternata* in the family *Pachastrellidae*. They were all collected in triplicate in the same location (site DR15), and also *P. ovisternata* was collected in triplicate in three other locations (DR10, DR9, and DR4) (Table S1). This sampling design allowed us to compare microbial community composition and function between sponge species and between locations.

Preliminary results showed significant similarities among the selected species; therefore to understand microbiome signatures of HMA deep-sea compared with that of shallow water sponges, we added 24 species of HMA sponges from deep-sea grounds of the Atlantic Ocean and the Caribbean, and 18 HMA species from shallow waters in the Mediterranean, Atlantic Ocean, and Caribbean, of those taxonomic orders that can be found in both deep and shallow waters (*Tetractinellida*, *Haplosclerida*, and *Chondrillida* from the class *Demospongiae*, and *Homosclerophorida* from the class *Homoscleromorpha*) (Table S2). The additional deep-samples were collected either by a rock dredge, a beam trawl, or a remotely operated vehicle, and manipulated in the same way as described above. Samples from shallow waters were collected by scuba diving and placed in separate plastic bags and brought to the shore where they were quickly rinsed, sliced, and stored in RNAlater and 96% ethanol (Table S2). We included from 1 to 3 replicates per species per site, making up 115 samples in this comparison. Of these, 33 species were available in our group from the same campaign (SponGES0617) and other surveys, and were prepared in the same way as described before using similar procedures to avoid contamination. Sequences from 11 species were downloaded from a public dataset that used the same primer set for the amplicon sequencing [16]. Collection details of these sponge species are available in Table S2.

### Community composition

To understand the microbial community composition and structure of deep-sea and shallow sponges (Table S2), we relied on the sequencing and analysis of the V4 hypervariable region of the 16S rRNA gene of prokaryotes. DNA was extracted from about 20 mg of tissue preserved in ethanol using the Dneasy Blood and Tissue kit (Qiagen, manufacturer’s protocol, July 2006 version). The tissue sectioned included cortex and mesohyl, just avoiding, or scratching, a thin layer of the outermost tissue to remove epibionts. The V4 region was sequenced using general microbial primers 515F-Y [38] and 806R [39], targeting both archaea and bacteria. DNA preparation, read processing, and taxonomic assignment were preformed following the same protocol as in Díez-Vives et al. [18]. Briefly, DNA amplification was done in duplicates with 28 PCR cycles, libraries prepared with the Nextera XT DNA Library Preparation Kit (Illumina Inc.), and next-generation, paired-end sequencing was performed at the Natural History Museum of London on a MiSeq System (Illumina) using v3 chemistry (2 × 300 bp). Samples were sequenced in two different MiSeq runs, but in the same machine. Our sequences and the ones downloaded from a public repository were combined in a single dataset and analyzed together bioinformatically to allow direct comparisons. Read processing of this combined dataset and taxonomic assignment followed the MiSeq SOP protocol [40] in Mothur (v.1.48.0) inferring amplicon sequence variants (ASVs), allowing one mismatch per 100 bp [41]. Chimeric sequences were removed using “chimera.uchime” command in mothur. ASVs were classified using the Silva database v.132, with a cutoff value of 80. The Silva 132 database places the family *Nitrosopumilaceae* within the phylum *Thaumarchaeota* (class *Nitrososphaeria*; order *Nitrosopumilales*); however, this has been reassessed, placing this family into phylum *Crenarchaeota* [42].

### Functional repertoire

Total RNA extraction and prokaryotic mRNA enrichment followed an adaptation of the protocol established by Díez-Vives *et al.* [24]. Total RNA was extracted from 50 mg of RNAlater preserved sponge tissue with TRIzol (Thermo Fisher Scientific, Waltham, MA, USA), performing physical disruption of tissue using a pestle for microcentrifuge tubes (micropestle, Eppendorf, Hamburg, Germany) attached to an electric drill. The RNA extraction was purified with a PureLink RNA Mini kit (Thermo Fisher Scientific), including Dnase treatment to remove contaminant DNA. Ribosomal RNA (rRNA) was depleted using a RiboZero kit (Illumina, San Diego, CA, USA) with a mix of probes targeting bacteria and plants, followed by NEBNext Ultra Directional RNA library construction. Sequencing was conducted on an HiSeq 4000 System (Illumina) with 150 bp paired-end chemistry (Centre for Genomic Research, University of Liverpool, UK) obtaining an average of 50 M reads/sample. We also used the raw metatranscriptomic reads from *P. angulospiculatus* (HMA sponge) and *H. caerulea* (LMA sponge), kindly provided by Dr Sara Campana and Dr Jasper de Goeij (Campana *et al.* under review), which were collected in shallow waters of the Caribbean to perform comparisons with our deep-sea sponges metatranscriptomes (Table S1). These samples were processed and analyzed following exactly the same methods and pipelines as in the present work, therefore a direct comparison could be done.

The reads were quality filtered with Prinseq lite v0.20.4 and contaminating rRNA reads were removed using SortMeRNA v2.1 [43] with the SILVA 119 Ref NR 99 database [44]. We used Kraken 2 [45] to remove eukaryotic reads from the dataset, which represented 9.33% of reads; however, most of the reads (i.e. 80%) were unable to be classified with this tool. The remaining reads were rarefied to 20 million reads/sample to reduce the database size and allow analysis with our computing infrastructure (Table S3). Reads were de novo assembled with the Trinity software v2.0.6 [46], using default settings for strand-specific, paired-end reads, and a minimum transcript length of 150 bp. Transcript abundance for each sample was estimated with RSEM v1.2.21 [47], which implements the Bowtie aligner [48], by mapping reads back to the reference metatranscriptome and calculating expected counts (number of reads mapped) and the normalized trimmed mean of M values (TMM). One individual, *P. ovisternata* DR10_477, showed signs of sample degradation, with low PCR amplification and low RNA amount, which resulted in a different sequence profile and was therefore excluded from comparative analyses.

The reference metatranscriptomes were uploaded to the IMG/M [49] for gene annotation [50-53]. Bacterial transcripts usually contain more than one ORF, in our case 36.6% of transcripts included a single protein, but the remaining transcripts presented from 2 to 12 proteins. To account for the expression of the annotated proteins individually, instead of the full transcript, we calculated the estimated counts proportional to the length of the peptide sequence. The estimated counts of the peptides were then transformed to transcripts per million (TPM). The expression of transcripts annotated as the same KEGG Orthology terms (KO) was added into an aggregated expression value.

For an overview of the function of deep-sea sponge microbiome, KOs were mapped into KEGG pathways and modules (groups of genes organized by steps in a metabolic pathway as defined by KEGG) using the on-line tool KEGG mapper [54]. Each module was further hierarchized into two higher levels of organization, as defined in the KEGG website, for modules (here called level B and Level C). Similarly, each pathway was further classified in two higher levels (here called level A and Level B). Due to the low variability between samples (see Results), general functions will be described using averaged expression values across all samples (i.e. aTPM). Whenever a KO was included in more than one pathway or module, its expression was added in all the pathways that were present. It is important to stress that most of the enzymes included in modules or pathways are involved in more than one cellular process (63.2% of the genes are included in more than one KEGG pathways); therefore, some of the general modules and pathways values reported can be overestimated. The number of times an enzyme is included in a reaction can be found in Tables S4 and S7. Shallow water sponge sequences were analyzed in the same way, therefore a direct comparison could be done.

### Statistical analyses

Total ASVs were rarefied before any analysis. When exploring only *G. barretti, G. pachydermata,* and *P. ovisternata* species, rarefaction was done to the minimum count number in these samples (i.e. 82 922). For the entire dataset (115 samples with all the additional species), this was rarefied to 20 000 counts (leaving only 5 samples bellow this threshold). Shannon index was calculated using rarefied samples, and this metric was compared among groups using analyses of variance (ANOVA). For beta diversity analysis, the relative abundances of the rarefied ASVs and the transcript abundance (trimmed mean of M-values, TMM) were log2 transformed prior to the calculation of Bray–Curtis dissimilarities. Homogeneity of Bray–Curtis dispersions were tested using ANOVA. Dissimilarity matrices were visualized using Principal coordinates analysis (PCoA) using “cmdscale” in vegan v. 2.6-4 [54] within R Statistical Software (v4.2.3; [55]), and differences between groups (species and locations) analyzed by permutational multivariate analyses of variance (PERMANOVA) using “adonis” and pairwise analysis. Shared and specific features were determined after filtering >0.01% relative abundance of ASVs and >1 TMM of transcripts. When analyzing the 44 additional species, alpha and beta diversity were studied in the same way but at the genus level, to reduce some variability associated with the different sequencing runs.

Differentially abundant ASV and differentially expressed genes were investigated with edgeR package v3.8.6 [56]. ASVs were filtered at 0.001% relative abundance, and 10 counts per million for transcripts, in at least 2 samples. Observed library size was normalized by applying scaling factors (TMM) computed internally by edgeR functions. ASVs or transcripts with an adjusted (i.e. Benjamini–Hochberg corrected) *P* value of less than 0.01 and 2-fold change were considered differential between groups. All visualizations were done using ggplot2 package [57] in R.

## Results

### Taxonomic composition of deep-sea HMA sponge microbiome

**Figure 2:**
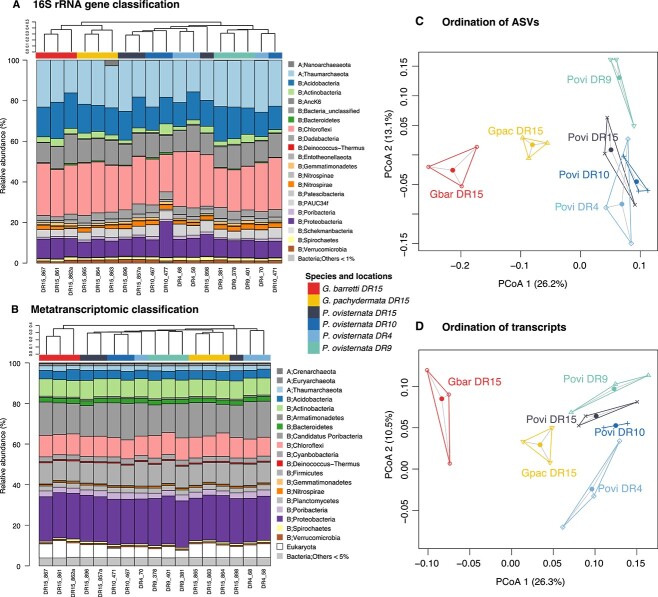
Sponge microbiome relative abundances of ASVs (A) and transcripts (B) grouped at phylum level, and phyla identified in both datasets share the same color coding, and shades of gray depict phyla that are only identified in either the ASVs or transcripts dataset; top dendrograms were based in the Bray–Curtis dissimilarities of sponge microbiome ASVs, and (C, D) principal coordinate (PC) ordination plots of Bray–Curtis dissimilarities of sponge microbiome ASVs (C) and transcripts (D); Gbar = *G. barretti*, Gpac = *G. pachydermata*, Povi = *P. ovisternata*. B = bacteria, A = archaea.

Sequencing of the region V4 of the 16S rRNA gene of *G. barretti*, *G. pachydermata*, and *P. ovisternata* resulted in an average read count of 131 757 ± 37 272, which represented from 6512 to 23 395 unique ASVs per sample. For the beta diversity, shared and differentially abundant ASVs analysis, we only considered ASVs showing at least 0.01% relative abundance in one of the samples; this left from 898 to 990 ASVs per sample but still represented from 83.8% to 94.4% of the total abundance. The microbial community was very similar among the three deep-sea species, dominated by the phylum *Chloroflexi*, with an average relative abundance (aRA) of 23.5%, including classes such as *Dehalococcoidia* (15.4% aRA) and SAR202 clade (14.6% aRA). This was followed by the phylum *Thaumarchaeota* (21.3% aRA), exclusively represented by the archaeal class *Nitrososphaeria*, and by the phylum *Proteobacteria* (9.3% aRA) with dominance of the class *Gammaproteobacteria* (5.7% aRA) (Fig. 2A and Table S4).

Regarding the RNA sequencing, samples had an average of 51 ± 6.8 million reads per sample. Raw reads presented an average of 1.9% of prokaryotic ribosomal RNA and 1% of eukaryotic rRNA, which was removed from the dataset. All samples were pooled together to generate a reference metatranscriptome of 5 118 275 transcripts including the expression of eukaryotic genes and all microbial members present among the samples. Transcripts with >500 nt length (1700341) were transcribed into 2 665 341 peptides, and 69% of these were taxonomically annotated, 1 708 990 peptides as prokaryotes and 131 522 as eukaryotes. Each sponge individual expressed an average of 1 414 561 ± 28 364 of the prokaryotic annotated peptides. The taxonomical classification of these transcripts revealed that 23.7%, on average, of the relative abundance of transcripts was classified as phylum *Proteobacteria*, 17% aRA as *Candidatus* Poribacteria and 12% aRA *Chloroflexi* (Fig. 2B). Archaeal transcripts only represented 3.6% aRA (1.9% aRA for phylum *Thaumarchaeota*; Table S5), which contrasts with the archaeal dominance in the amplicon sequencing results (second in abundance after phylum *Chloroflexi*). This could show that the archaeal community is not functionally active, but it is also likely that archaea are underrepresented in the protein databases, hence biasing the annotation (IMG included 94 554 bacterial genomes and 2150 archaeal genomes). Other phyla showed discrepancies in relative abundances among the methods (e.g. *Nitrospirae*), but overall, the microbial taxonomical composition was highly similar across individuals in both datasets (i.e. amplicon sequencing and metatranscriptome, Fig. 2A and B).

### Drivers governing the deep-sea sponge microbiome

**Figure 3:**
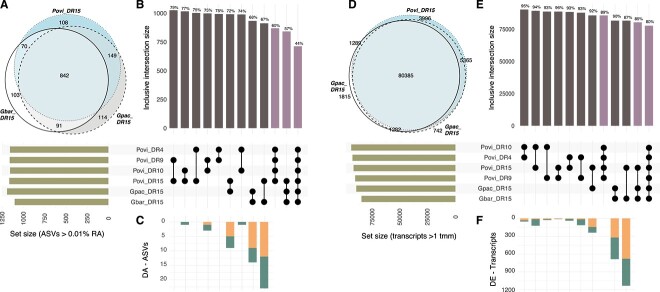
Shared ASVs (A–C) and transcripts (D–F) between sponges species and among locations; (a, D) Venn diagrams showing intersecting sets (ASVs and transcripts) of the three sponge species in location DR15, and (B, E) upset plots showing inclusive intersections sets (connecting dots) of all samples, and top barplots showing size and percentages of the shared features by pairs (gray) or multisample comparisons (purple); left bars indicate the set size of each sample, and (C, F) stacked barplots show the number of differentially abundant ASVs and differentially expressed transcripts of each pair-wise comparison, and teal and orange areas represent the number of up- and downregulated features (details in Table S6); Gbar = *G. barretti*, Gpac = *G. pachydermata*, Povi = *P. ovisternata*.

To identify the role of sponge identity and location shaping the composition of the sponge-associated microbial communities in the *Geodia* and *Pachastrella* species, we studied whether these factors were shaping the microbial community by exploring shared vs. specific features, and the microbiome beta diversity. We identified high numbers of shared ASVs (67%–72%) between species, representing 94%–96% of the relative abundance of the community, and from 74% to 79% shared ASVs between locations (Fig. 3A). Similarly, between 87% and 92% of shared transcripts were found between species, and 96% between locations (Fig. 3B). Among the differential features, we detected few differentially abundant ASVs (maximum of 23, representing 0.23% of ASVs, between *G. barretti–P. ovisternata*, Table S6, Table S10), and differentially expressed transcripts (maximum of 1132, representing 4% transcripts, between *G. barretti–P. ovisternata*; Figs S1 and S2, Text S1, Table S6, Table S11).

Even with this large similarity in the microbiomes, the microbial community composition still reflected differences across species at the ASV level. The dendrograms based on the ASVs relative abundances maintained a larger similarity between *Geodia* spp. compared with *P. ovisternata* (Fig. 2A), while according to transcript expression levels, *G. pachydermata* was more similar to *P. ovisternata* than to *G. barretti* (Fig. 2B). Similarly, in the ordination analysis of the ASVs, the first axis separated the sponge species equally, whereas the second axis roughly separated locations (Fig. 2C). PERMANOVA tests confirmed the differences between the species in site DR15, and the analysis on locations for *P. ovisternata* only identified DR9 as significantly different from DR10 and DR4 (Table S7). Ordination of samples based on transcript abundances, and correspondent PERMANOVA tests, found differences between *G. barretti* and the other two species but did not find differences between *G. pachydermata* and *P. ovisternata* (Fig. 2D). Among locations, only DR4 samples were significantly different from DR9 and marginally from DR15 (Table S7).

### Compositional variability in deep and shallow-water sponge microbiomes

**Figure 4:**
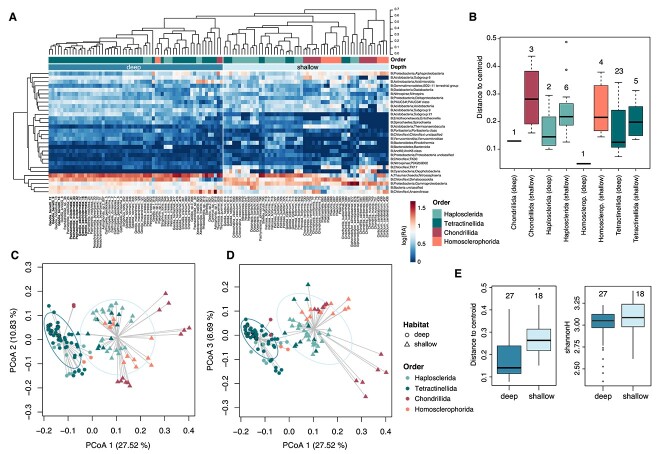
(A) Heatmap of sponge microbiome relative abundances (ASVs grouped at class level) among four orders of deep and shallow HMA sponges, and top dendrogram is based on the bray–Curtis dissimilarity of microbiome relative abundances (ASVs grouped at genus level); (B) variability of sponge microbiomes grouped by sponge order and habitat depth, and numbers above boxplots are the number of sponge species included, and (C, D) PC ordination plots of bray–Curtis dissimilarities of microbiome relative abundances (ASVs grouped at genus level) showing first and second PC axis (C) and first and third PC axis (D); the second combination of axis helps to visualize differences between orders of the shallow species; the three deep-sea tetractinellid species of this study are highlighted by a black outline; (E) variability of bray–Curtis dissimilarities (left) and Shannon diversities (right) of sponge microbiomes samples across habitat depth; B = bacteria, A = archaea.

Given the remarkable similarity in the microbiome of these three tetractinellid sponges, we tested whether the tetractinellid microbiome was inherently more homogeneous than that of other sponge orders (*Haplosclerida*, *Chondrillida*, and *Homosclerophorida*) regardless of the habitat, or whether the high similarity in their microbial composition was ruled by the environmental constraints of the deep-sea habitat. We included 45 HMA species from shallow and deep waters that comprised from 10 550 to 366 212 counts per sample, representing from 290 to 21 592 unique ASVs. We found an exceptional similarity among deep-sea sponge species regardless of their taxonomic affiliation (Fig. 4A), with very high abundances of *Nitrosphaeria* (*Thaumarchaeota*) and *Dehalococcoida* (*Chloroflexi*), and absence of *Cyanobacteriia* (Table S8). Shallow species of these four sponge orders presented larger differences in microbial composition resulting in a higher variability than their deep-sea counterparts (*P* < 0.001) and larger diversity (*P* = 0.003) (Fig. 4B and E).

The microbiome of the shallow water species was species-specific (based on the genus level of microbes), particularly in *Chondrillida* and *Homosclerophorida*, and to a certain extent also in *Haplosclerida* and *Tetractinellida* (Figs 4C and D and S3). In the deep-sea environment though, *Chondrillida* was still the most dissimilar order in the clustering (Figs 4 and S3), but the other three orders were tightly grouped together, showing remarkable similarity among species, especially in *Tetractinellida*, and most notable among *G. barretti, Geodia nodastrella, Pteretis nodulosa, P. ovisternata, G. pachydermata,* and *Neoschrameniella bowerbanki* (Figs 4C and D and S3). In fact, pairwise ANOVA comparisons (for samples with replicates) detected 36 species pairs that were not significantly different (*P* > 0.01), many of them collected in different locations, while among shallow water species, all pairs were significantly different (*P* < 0.001, Table S9).

### Functional profiles of deep and shallow-water sponge microbiomes

**Figure 5:**
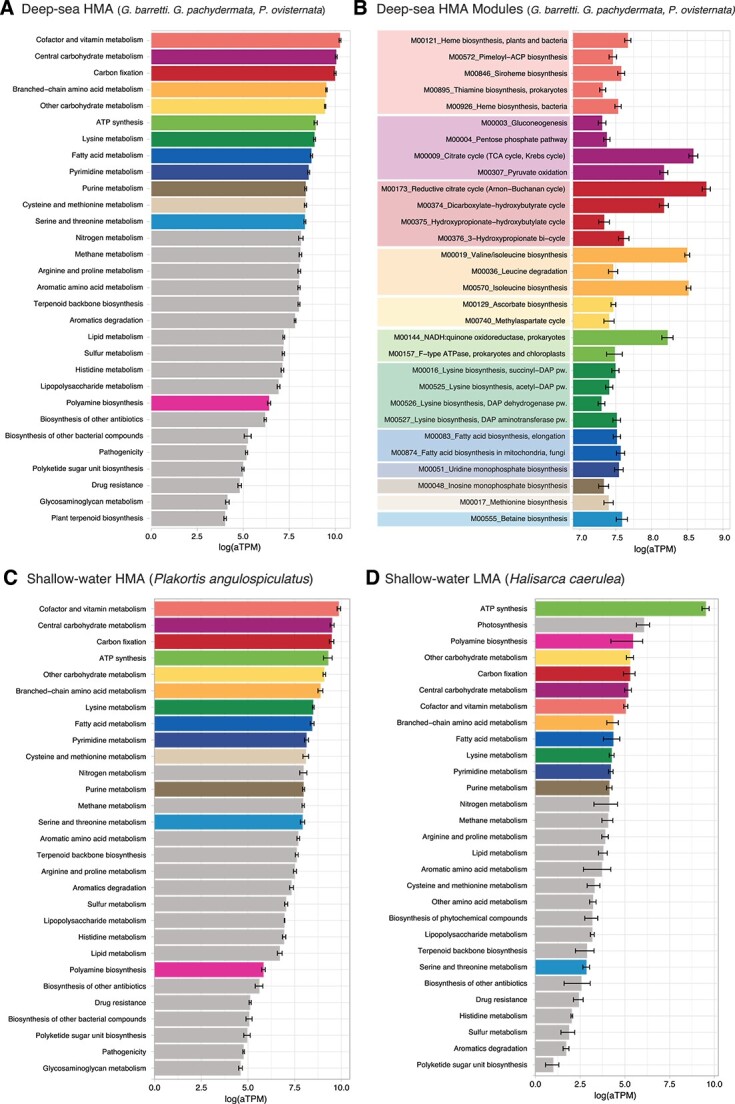
(A) Expression of KEGG modules grouped by reference pathways for the three deep-sea HMA sponges, and most abundant pathways among the species are colored to ease comparisons between plots; (B) individual modules within the top 12 reference pathways shown in panel a for the three deep-sea HMA sponges, and (C, D) reference pathways for the shallow-water HMA sponge (C) and the shallow-water LMA sponge (D).

**Figure 6:**
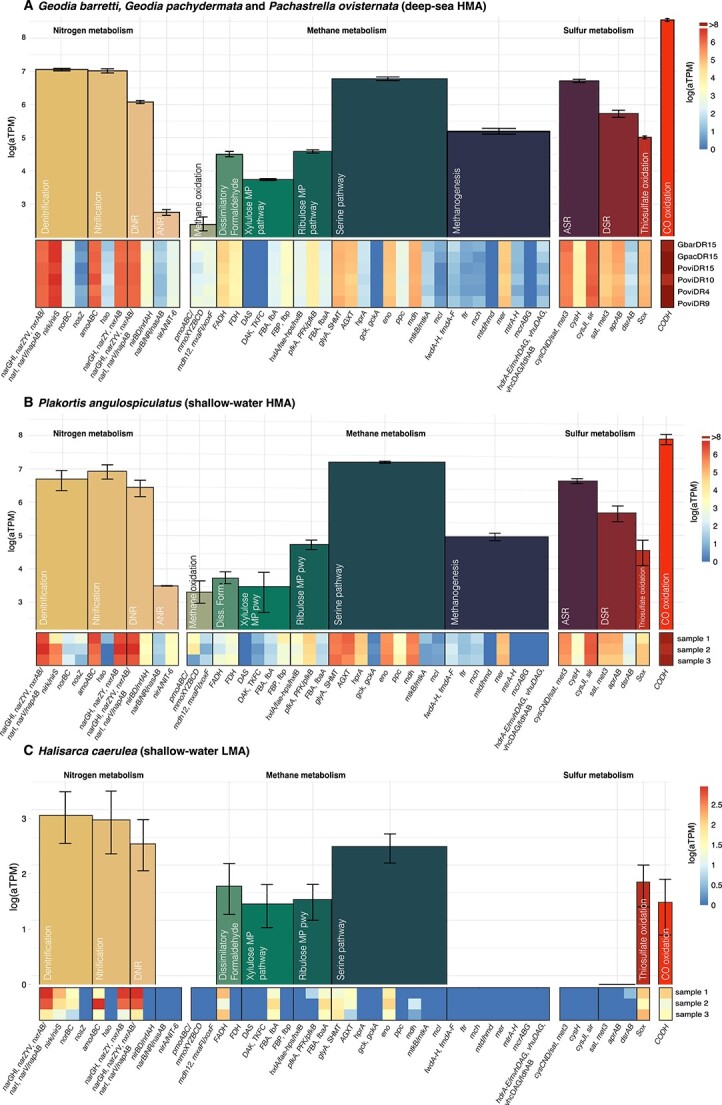
Averaged sponge microbiome gene expression values of KEGG modules within energy metabolism and their constituent reactions across three deep-sea HMA (A), a shallow-water HMA (B), and a shallow-water LMA (C) sponges; top bars show KEGG module expression and bottom heatmap constituent gene expression; DNR = dissimilatory nitrate reduction, ANR = assimilatory nitrate reduction, ASR = assimilatory sulfate reduction, DSR = assimilatory sulfate reduction.

In general terms, deep-sea HMA sponges also presented highly similar functional activities of the symbiont communities, with comparable expression values and completeness of the modules (Figs 5A and S4 and Tables S12 to S14). For shallow water sponges, a total of 810 763 transcripts were present in *P. angulospiculatus*, and 600 037 transcripts in *H. caerulea*, which were annotated in IMG/M. Annotation recovered 48 309 and 4589 transcripts to prokaryotic KO terms, respectively, which were grouped in modules and compared with our deep-sea datasets. The shallow water HMA sponge, *P. angulospiculatus*, resembled the expression patterns of deep-see sponges to some extend (Fig. 5C); however, in the shallow water LMA sponge, *H. caerulea*, photosynthetic transcription made up a large proportion of the symbiont transcriptional profile, as well as polyamine biosynthesis and ATP synthesis (Fig. 5D). Nitrogen and methane metabolism were highly expressed in both HMA and LMA sponges, but sulfur metabolism was less abundant in the LMA sponge than the HMA sponges, with only thiosulfate oxidation moderately expressed (Figs 5 and 6). In both deep and shallow HMA sponges, symbionts dedicated a large part of their DNA transcription to genes necessary for the synthesis of cofactors and vitamins, though much less in the shallow-water LMA sponge (Fig. 5). Other high expressed functions among all species were related to central carbon metabolism, carbon fixation, amino acid biosynthesis, and fatty acid biosynthesis (Fig. 5 and Text S2).

Among the most expressed individual metabolic modules in our three deep-sea species (Text S2), we found carbon degradation pathways such as tricarboxylic acid cycle (TCA; 5343 atpm) or Pentose phosphate cycle (1573.5 atpm; Fig. S5A, Text S2). Besides glucose, sponge symbionts used a variety of additional carbon sources (i.e. galactoside, fructoside, xyloside, and rhamnoside; Fig. S5B, Text S2). Gene expression for autotrophic carbon fixation was also high for the different cycles (Fig. S5C), with the complete expression of the reductive citrate cycle (Arnon-Buchanan cycle or rTCA, 6319 atpm, being also the highest single module expressed in the entire transcriptome), and complete Reductive pentose phosphate cycle (Calvin cycle, 1306 atpm). The remaining modules were incomplete (Figs S4 and S5, Table S14). All modules associated with nitrogen metabolism, except for nitrogen fixation, were complete and highly expressed (i.e. nitrification with 1130 atpm, denitrification with 1176 atpm; Fig. 6, Text S2, and Table S14). Ammonium transporter (Amt) was the most expressed type of transporter, indicating the great use of this molecule to supply nitrogen demand for nitrification (1535 atpm, Table S15). The second enzyme involved in nitrification, hydroxylamine dehydrogenase (hao), however, was expressed in much lower rates (4.7 atpm). Similarly, denitrification was highly expressed in general, but the last two reactions of this process (reduction of NO to N_2_O, and finally to N_2_) were expressed in lower levels (up to 9.6 atpm; Fig. 6A, Table S15), indicating a possible accumulation of nitric and nitrous oxide (NO) during denitrification. In the shallow water LMA demosponge, the expression of *nosZ* was completely absent (Fig. 6C), and the remaining reactions presented the expression of some genes but not entire protein complexes, therefore listed as incomplete (Fig. S4). Modules related to methane metabolism (i.e. methane oxidation and assimilation of formaldehyde by the serine or ribulose monophosphate pathways) were complete, although methane oxidation was not high if only confirmed methanotrophs were considered (Text S2). Methanogenesis was not complete. We also found the expression of complete modules related to sulfur metabolism, CO oxidation via carbon monoxide dehydrogenase, and phosphonate utilization as potential source for growth in conditions of limiting inorganic phosphorous (Pi) concentrations (Fig. 6A, Text S2).

Many functions related to symbiotic lifestyles in the deep-sea sponges were also highly expressed (Text S3, Tables S16 to S18) such as ATP binding cassette transporters pathways (9568 atpm), quorum sensing pathway (21 428 atpm), secretion systems Type II (2767 atpm), eukaryotic-like repeat containing proteins (i.e. 9906 TPR transcripts at 3138 atpm), cell–cell adhesion molecules (cadherins at 1426 atpm), mobile genetic elements (7567 atpm), restriction-modification systems (1806 atpm), and CRISPR-related transcripts (361 atpm) (Text S3, Tables S19 to S22). Finally, among the individual genes (KO terms) with highest expression (Fig. S6, Text S4), we found several genes involved in the regulation of the microbial community, including three proteins related with peptide/nickel system for antibiotic production (8934 atpm), and the chaperonin GroEl (3810 atpm; Text S4), with proposed roles in microorganisms–host interactions.

**Figure 7:**
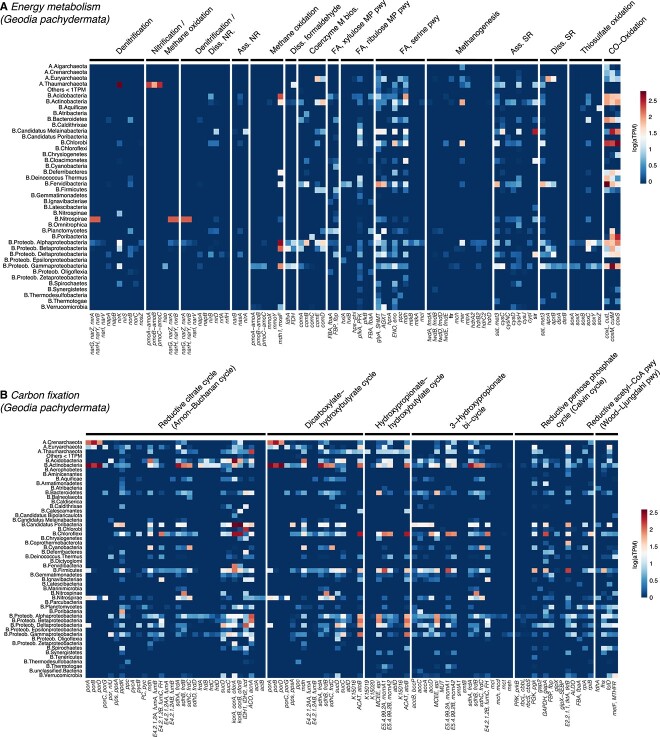
Averaged sponge microbiome gene expression values of KEGG modules, split by microbial phyla, involved in energy metabolisms (A) and in carbon fixation (B) for *G. pachydermata*; Diss. NR = dissimilatory nitrate reduction, Ass. NR = assimilatory nitrate reduction, Ass. SR = assimilatory sulfate reduction, Diss. SR = dissimilatory sulfate reduction.

Half of the genes were common to more than 12 phyla, which is expected in widely distributed functions (i.e. essential functions); however, quantifying redundancy for specific metabolic reactions is complex. While some reactions were dominated by one group, such as the high expression of phylum *Thaumarchaeota* in gene *amoCAB*, others genes were expressed by several microbial taxa (Fig. 7A, Fig. S7, Fig. S8, Text S5). The use of different enzymes performing equivalent reactions (i.e. *nirk*/*nirS, napA*/*narG*), however, was not evident, since there was a preference of one enzyme over the alternative ones. This preference was the same in all three species, probably due to the large similarity of the microbiome across species.

## Discussion

### Microbial assemblages in deep-sea sponges

The sponge species used in this work, *G. barretti*, *G. pachydermata*, and *P. ovisternata*, which are common and abundant deep-sea tetractinellids restricted to the North Atlantic Ocean and often in sympatry [58, 59], presented a remarkable similarity in the composition and structure of their microbiome. They are HMA sponges, with body tissue containing up to 10^11^ microbes per cubic centimeter [33, 60]. Their microbiomes were dominated by *Chloroflexi*, *Thaumarchaeota*, *Acidobacteria*, *Actinobacteria*, and *Proteobacteria* (Fig. 2), which are classical components of HMA species [34, 61-63]. Marine demosponges are known hosts of symbiotic *Thaumarchaeota* [27, 28, 64-66] that are the actors in ammonia oxidation [67], and most also host bacteria affiliated with known nitrite-oxidizers in high numbers, particularly members of the genus *Nitrospira* [14, 23, 33, 68]. In our sponges, however, the relative abundance of *Nitrospira* was residual (Table S4), although they were active members in the nitrogen cycle. There was some discrepancy between the present microbial community (amplicon sequencing) and the active community (metatranscriptomics), where abundant groups were *Thaumarchaeota* and *Chloroflexi*, but most active members were *Proteobacteria* and *Poribacteria*, the latter has been suggested to be active only in the host context but not in the sea water [13].

Generally, sponge identity is a major driver of the symbiotic community composition [12, 14, 16, 69]; however, the extreme but largely stable environmental conditions of the deep-sea habitats [70, 71] may play a fundamental role restricting and homogenizing the composition and function of the sponge microbiome. Microbiome composition (at ASV level) was most similar between the *Geodia* spp., belonging to the family *Geodiidae*, than to *P. ovisternata* within family *Pachastrellidae* [59]. These two families may have diverged around 133 Ma [36], and perhaps, such phylogenetic and temporal divergence could be the main factor driving the differences, although small, in the composition of their microbiome, given that they live in sympatry. However, such slight differences were not retained at the functional level, since *G. pachydermata* and *P. ovisternata* displayed more similar transcriptional profiles (Fig. 2). The extreme similarities in the microbiome assemblage and function of these coexisting sponges (site DR15) might be explained by local restrictions, with only small differences expected from physiological differences between the sponge species. Across locations, environmental conditions were rather stable, with only sponges from El Corbiro Canyon (DR4) subjected to slightly higher saline waters (also see [30]), which could explain subtle differences in the composition and functionality of samples from DR4 to adapt to the local conditions (Fig. 1C). Similarly, Busch and collaborators [30] compared the compositional differences between the microbiomes of LMA fan-shaped sponges from this same area, and found that they were most likely driven by differences in temperature and salinity, which are, in general, the major drivers of compositional differences of deep-sea sponges [37].

### Stability of the deep-sea microbiome

To understand the magnitude and factors driving the high similarity observed in the microbiome of our *Tetractinellida* species, we compared our samples with other sponge orders present in both deep-sea and shallow-water habitats. The microbiome of deep-sea tetractinellids has been thoroughly described in the last few years given their abundance in deep-sea waters [27, 28, 37, 72], with rather similar microbial signatures regardless of their geographic dwelling [27, 28, 37, 72]. However, we believe that the host taxonomic breadth, replication, and sequencing approach was not ideal to extract sound conclusions about their compositional patterns, and direct comparisons between deep-sea and shallow water sponge microbiomes of the same orders are necessary. In our study, which contains the largest taxonomic and geographical range to date for HMA deep and shallow sponges, there was still an exceptional similarity (at genus level) among the microbial communities of deep-sea sponge across sponge species, which mirrors the lower variability of free-living marine microbial communities in deep-sea waters than those of shallow waters [73-76]. In this analysis, 35 pair comparisons between deep-sea species (including *G. barretti, G. pachydermata,* and *P. ovisternata*) could not be distinguished by their microbiome composition, while all pairs from shallow species were significantly different. We concluded that shallow-water sponges, belonging to the same orders than the deep-sea sponges but subjected to larger fluctuations in environmental conditions, presented a more variable and diverse community of symbiotic microbes, showing traditionally described species-specificity patterns [12, 16]. However, these patterns were greatly challenged by the extreme homogeneity in the microbiome of deep-sea sponge species, regardless of their phylogenetic relationships (Fig. S3). The limiting environmental drivers of host–symbiont interactions in deep-sea species seem to dominate over the potential restrictions imposed by the diversity in the immune systems governing species-specific symbiont–host relationships in sponges [77, 78].

Previous works have shown that within geographic sites, different sponge host species harbor divergent microbiome composition and function, as a mechanism for alleviating resource competition and for accessing novel resources [79, 80]. In the case of these deep-sea sponges, composition and function were highly similar, possibly showing high specialization to this environment, and also stability of resources promoting lack of competition among microbial members. A recent publication indicates that the strong evolutionary selection for ecological convergence across sponge lineages could also cross the barrier of phyla and affect other organisms such as corals, since large similarities in the microbiome functionality of corals and sponges were found in the deep-sea environment [26].

### Functional diversity of deep-sea and shallow-water sponge microbiomes

In sponges, much of the transcriptional activity of the symbiont communities are devoted to amino acid, energy, carbohydrate, and nucleotide metabolisms, as well as metabolism of cofactors and vitamins, regardless of their host habitat, phylogeny, or physiological status [22, 25, 32, 81]. To understand whether deep-sea sponges diverge from this general pattern, we compared our datasets of deep-sea HMA sponges with both HMA and LMA shallow sponges (*P. angulospiculatus* and *H. caerulea*, respectively). Both deep and shallow-water HMA sponges presented similar symbiotic functional activities (Fig. 5), with cofactor and vitamin metabolism dominating the transcriptional profiles, which are not only crucial for many other metabolic pathways but are also known to be beneficial for the sponge host [1, 25, 32, 63, 67, 82-84]. In turn, in the shallow water LMA sponge, the symbiont photosynthetic transcription dominated, which is central for the energy acquisition of the microbiome of other shallow-water LMA sponges [10, 22, 85, 86]. Nitrogen, sulfur, and CO oxidation were highly expressed in both our deep-sea and shallow HMA sponges, as reported before in other deep and shallow HMA sponges [32, 86, 87], but sulfur metabolism was almost absent in the LMA shallow water sponge. The fatty acid and amino acid biosynthetic pathways were also highly expressed across all our samples, indicating that sponges heavily rely on their microbiome for the biosynthesis of these monomers [34, 88, 89], for which host transcripts usually include only catabolic reactions or incomplete biosynthesis pathways [25, 90, 91]. Moreover, many features common to host-microbiome systems such as mobile genetic elements, chaperonin GroEL, ATP binding cassette transporters, microbial secretion systems, polyketide synthases, or Eukaryotic-like repeat containing proteins were expressed in high numbers, suggesting active interactions within the sponge environment (Text S3).

From recent studies, we know that in *G. barretti*, the microbiome is actively involved in the metabolism of C and N compounds, remarkably impacting the holobiont nutrition and expanding their metabolic capacity [33, 60, 88, 92]. In our analysis, carbon degradation pathways were highly expressed, indicating a large and active community of aerobic and heterotrophic microorganisms in these sponges, which can even contribute up to 87% of the total DOM assimilation by the sponge holobiont [10]. The oxygen-sensitive reductive TCA (rTCA), which is more energy-efficient than oxygen-tolerant cycles such as Calvin cycle (CBB), presented the highest expression in our samples and appears to be the main carbon fixation pathway at deep-sea vents where oxygen is depleted [93, 94]. The remaining modules of carbon fixation were incomplete, similar to what occurs in MAGs from other sponges [62, 66]. The lower expressed Calvin cycle has been identified in chemoautotrophs (e.g. sulfur, ammonia, iron oxidizing bacteria) including free-living and deep-see invertebrate symbiosis, in which CO_2_ fixation by the Calvin cycle provides the invertebrate host with a source of organic carbon [95–97]. Both rTCA cycle and CBB cycle can operate in the sponge environment and can be complementary in times when sponges are not pumping and the mesohyl turns anoxic [86, 98, 99], which often occurs in HMA deep-sea sponges [60, 100]. *Geodia barretti* controls the oxygenation levels of its tissue by performing pumping arrest, which facilitate the co-existence of aerobic and anaerobic symbionts [100]. These microenvironments also allow co-existence of anaerobic (denitrification) and aerobic (nitrification) within the same individual. *Geodia barretti* is able to release nitrate rather than ammonium, interpreted as net nitrification [60], which is in agreement with the full completion and high expression of all nitrogen modules in our deep-sea sponges, except for nitrogen fixation. Ammonia oxidation seems to be largely performed by archaea in all three species, as previously reported in *G. barretti* [33, 101]. These archaea were classified within families *Nitrosopumilaceae* and Ca. Nitrososphaeraceae, which are known as common, abundant, and highly active prokaryotic symbionts of deep-sea sponges across the globe [28, 37, 102, 103]. Fixed carbon fuelled through ammonia oxidation reductive power in deep-sea sponges, seem to be equivalent to the photosynthates obtained by cyanobacterial symbionts in shallow water sponges [104]. The rare expression of NO reduction genes (*nosZ*, only by *Alphaproteobacteria*) during denitrification in our deep-sea sponges could result either in the release of NO into the surrounding seawater or the presence of alternative routes producing final N_2_ and O_2_ bridging major pathways [6, 23, 31, 83, 105-108]. Alternatively, the accumulation of NO could be involved in the regulation of physiological processes such as larval settlement, aquiferous system development, or regulation of body contractions [109-112]. Modules for methane oxidation and ribulose monophosphate pathway were complete but relatively low, indicating a possible but moderate use of methane as a source of energy and carbon by MOX symbionts in our species compared with sponges from deep asphalt seeps [35].

In our deep-sea sponges, we found high redundancy in many functional pathways, as described before in the sponge microbiome [83, 113], which may share a set of core functional genes rather than a common set of taxa [114-117]. The number of microbial members expressing the same gene within a sponge indicates that the sponge environment is a stable ecosystem with large functional redundancy. Similarly, the low evidence of different enzymes performing equivalent reactions across the species was related to the high similarity of the microbiome but could also suggest a lack of competition among the microbial members of different sponge species coexisting in deep-sea environments.

## Conclusions

A large number of putative symbiotic functions have been hypothesized from taxonomic or metagenomic data including necessary tools for a symbiotic lifestyle; however, evidence to validate potential symbiont physiologies is comparably rare, even less in deep-sea sponges. By studying the complete expression of all sponge-associated microbes, our study identified an surprising similarity in the microbiome composition and function of deep-sea sponge species that is unknown for shallow-water sponges. Interestingly, the microbial functioning of the microbiome showed a larger similarity between species from different genera (*G. pachydermata* and *P. ovisternata*), than for sponges of the same genus (*G. pachydermata* and *G. barretti*). Moreover, the microbiome composition of several deep-sea sponges could not be distinguished at the genus level, while all shallow-water species compared presented classic species-specificity. It could be speculated that, unlike shallow water equivalents, where the microbiome acts as a mechanism for ecological diversification within sponges, in deep-sea sponges, the microbial composition and functionality has converged to adapt to an extremely restrictive environment with the aim of exploiting the available resources. More notably, this similarity could possibly be extensive to other deep-sea organisms such as corals. We also observed that the active microbial communities were formed by many redundant members performing the same functions, and scarce use of alternative enzymes, pointing to the stability of resources in the deep-sea reducing competition among microbial members.

## Supplementary Material

RNA_Supplementary_Figures_reviewed_wrad030

RNA_Supplementary_Tables_reviewed_wrad030

RNA_Supplementary_Text_reviewed_rev_clean_wrad030

## Data Availability

Raw sequence data for all sponge species studied here are available from the NCBI SRA under project PRJNA988918. The combined metatranscriptome assembly *of G. barretti, G. pachydermata*, and *P. ovisternata* samples is available from IMG/M under ID 3300042672. Data analysis scripts are available in the GitHub repository https://github.com/cdiezvives/deep-sea-sponge-ISME-2023.
