# Supplementary material for: High compositional and functional similarity in the microbiome of deep-sea sponges
Source: ISME J. 2024 Jan 12;18(1):wrad030. doi: 10.1093/ismejo/wrad030 (PMC10837836; doi:10.1093/ismejo/wrad030)
Supplement: RNA_Supplementary_Figures_reviewed_wrad030 [file rna_supplementary_figures_reviewed_wrad030.pdf]

SUPPLEMENTARY FIGURES

High compositional and functional similarity in the microbiome of deep-sea sponges

Cristina Díez-Vives[1,3], Ana Riesgo[2,3]

[1] Department of Systems Biology, Centro Nacional de Biotecnología, c/ Darwin, 3, 28049, Madrid, Spain

[2] Department of Biodiversity and Evolutionary Biology, Museo Nacional de Ciencias Naturales (CSIC), c/José Gutiérrez Abascal 2, 28006, Madrid, Spain

[3] Department of Life Sciences, The Natural History Museum, London, SW7 5BD, UK

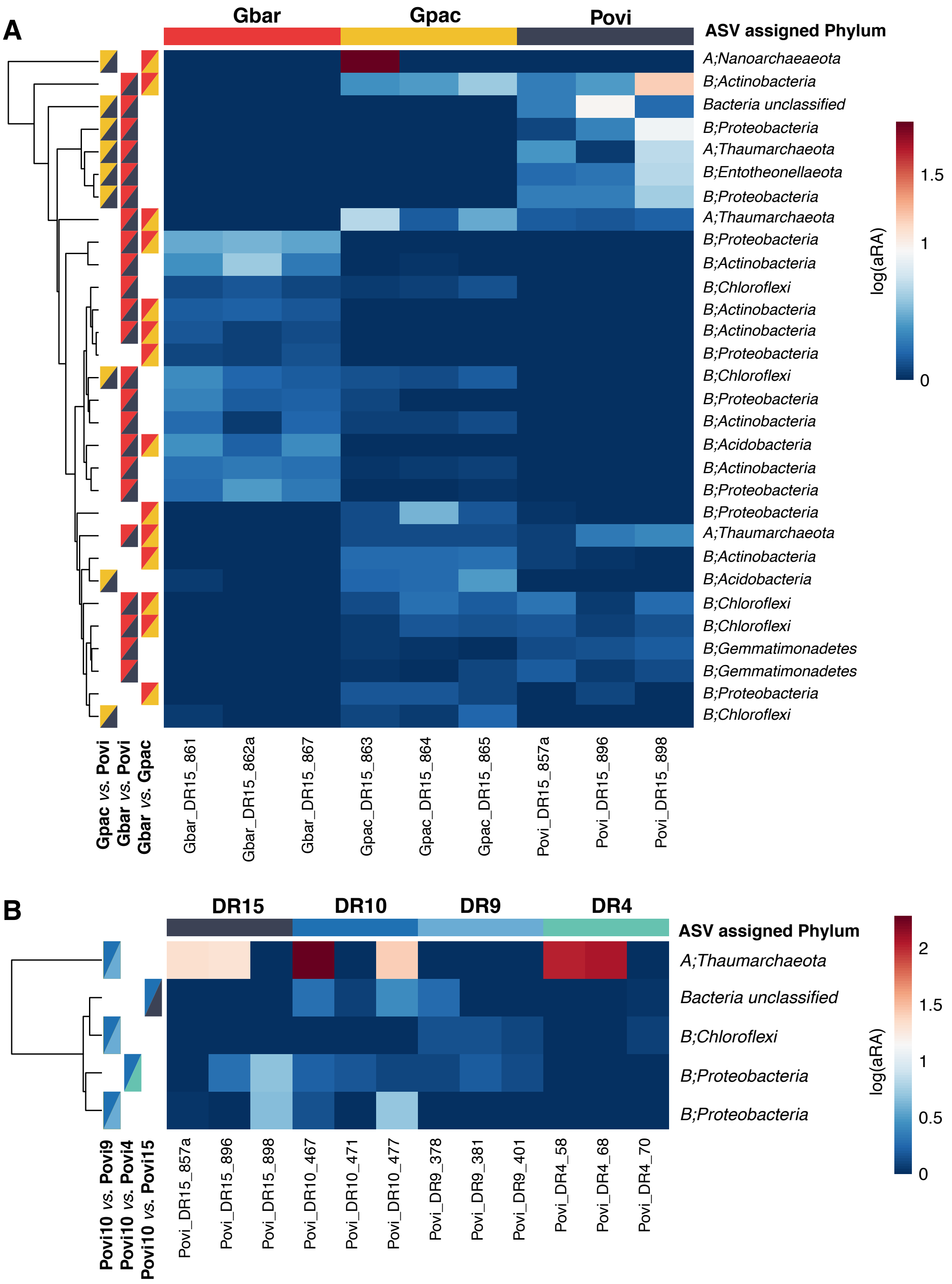

Fig. S1. Differentially abundant (DA) sponge microbiome ASVs detected between species in site DR15 (**A**), and between locations for *P. ovisternata* (**B**). Right side labels show the ASV classification at phylum level. Left side squares denote whether a significant pairwise difference was detected between sponges species or locations for an ASV. Squares contain annotation colors assigned to each sponge species as observed at the top of the heatmaps. Gbar = *G. barretti*, Gpac = *G. pachydermata*, Povi = *P. ovisternata*. B = Bacteria, A = Archaea. DR indicates sampling sites for *P. ovisternata*.

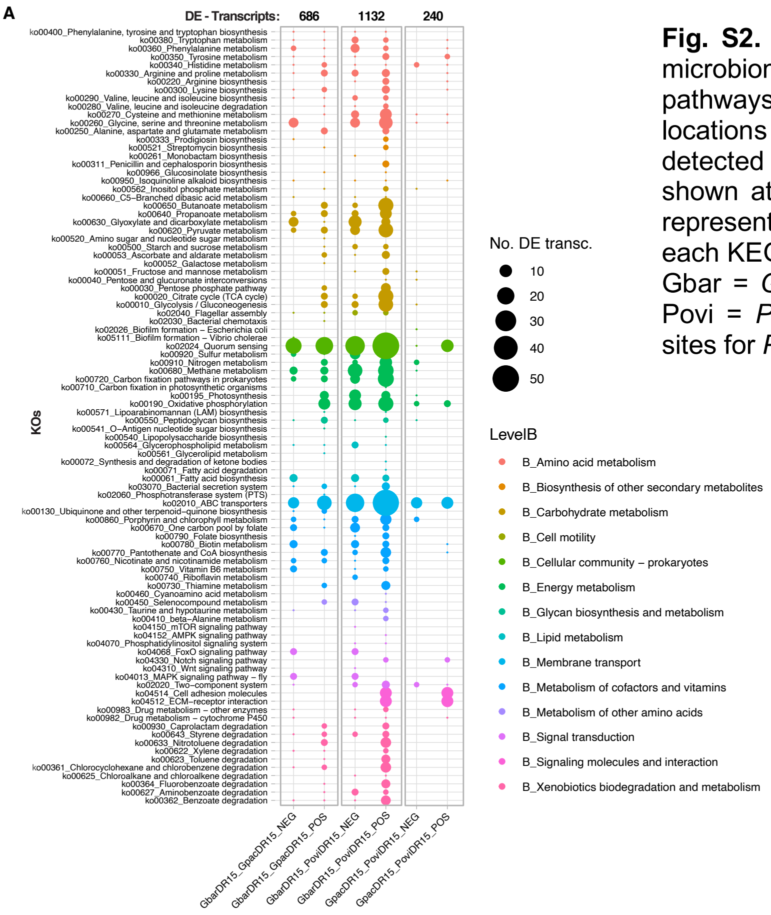

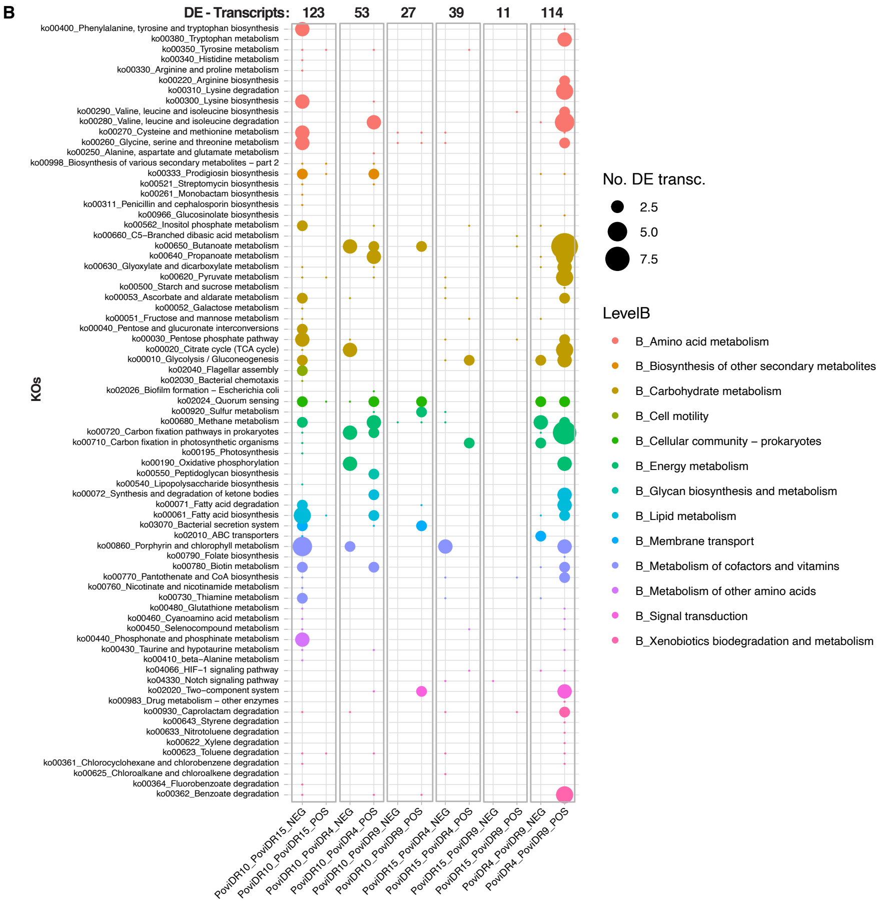

Fig. S2. Differentially expressed (DE) sponge microbiome transcripts grouped by KEGG pathways, between sponge species (**A**), and locations (**B**). Number of DE transcripts detected for each pairwise comparison are shown at the top of each plot. Size of circles represent number of DE transcripts included in each KEGG pathway. Gbar = *G. barretti*, Gpac = *G. pachydermata*, Povi = *P. ovisternata*. DR indicates sampling sites for *P. ovisternata*.

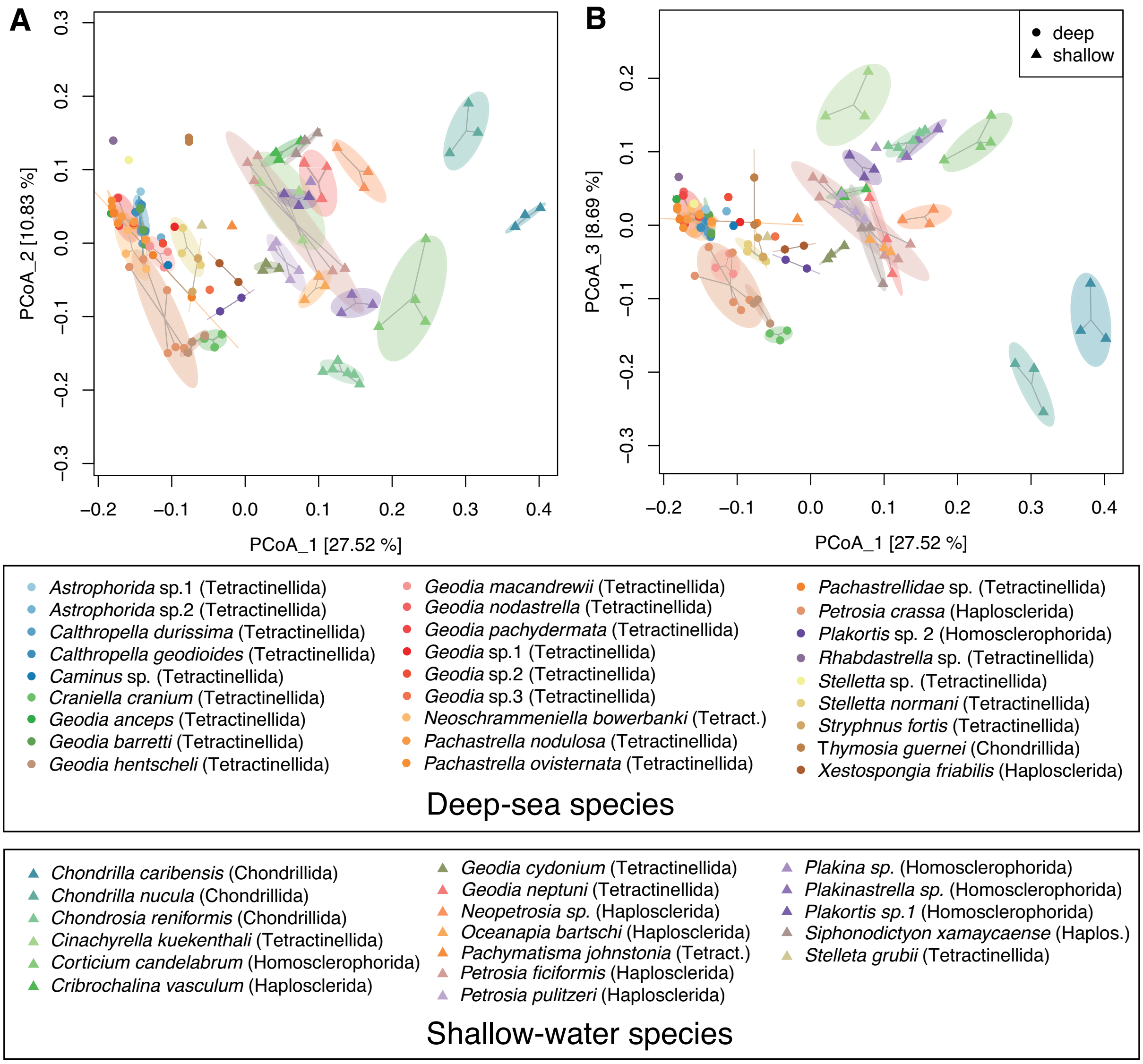

Fig. S3. Principal Coordinate (PC) ordination of Bray-Curtis dissimilarities (at genus level) between sponge microbiomes in deep-sea and shallow HMA samples for the four selected sponge orders, coloured by the sponge species. **A.** First and second PC axis. **B.** First and third PC axis. The second combination of axis helps to visualize differences between orders of the shallow species.

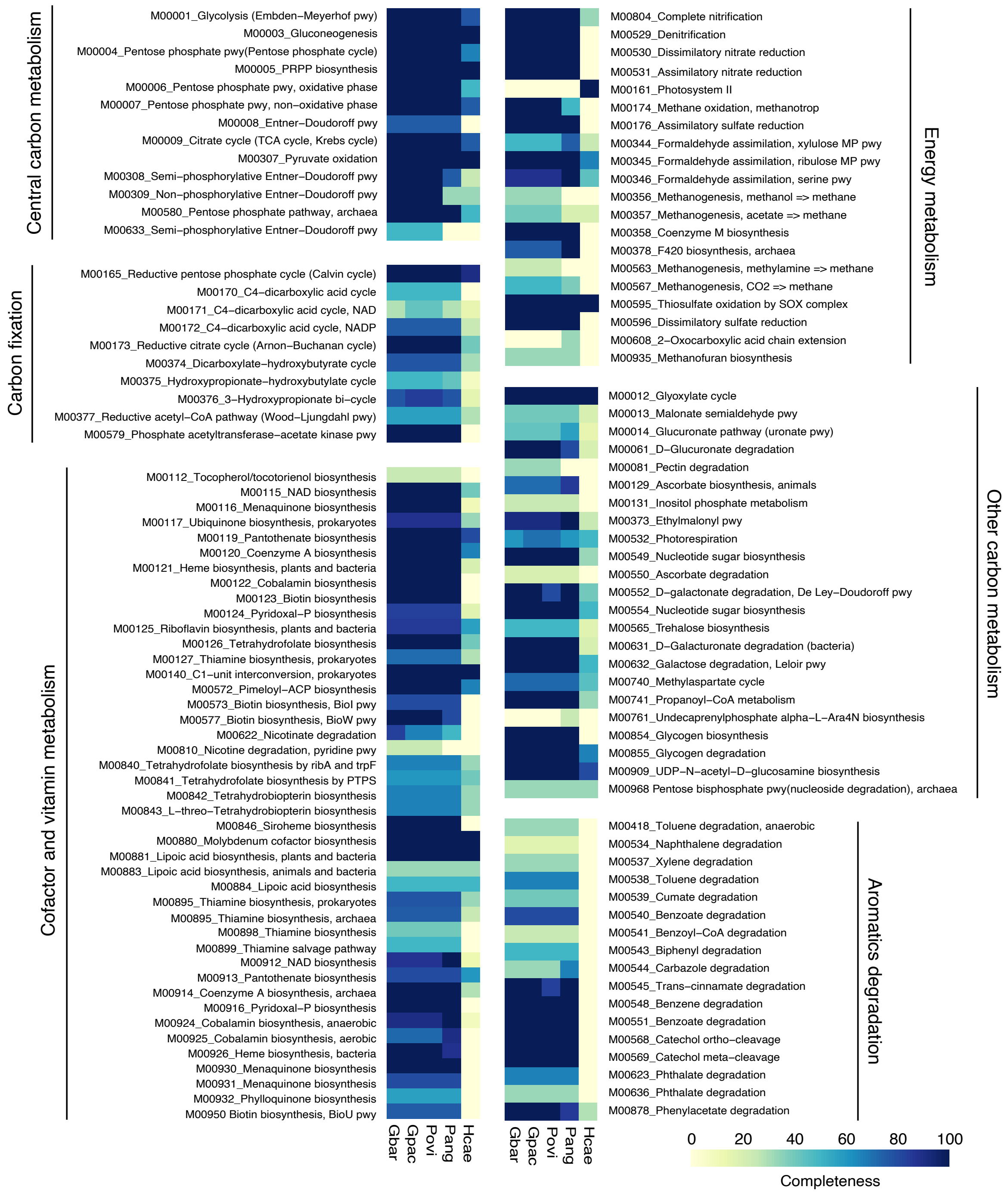

Fig. S4. KEGG module completeness by sponge microbiomes in deep-sea and shallow HMA , and shallow LMA sponge species.

Gbar = *G. barretti*, Gpac = *G. pachydermata*, Povi = *P. ovisternata*, Pang = *Plakortis angulospiculatus,* Hcae = *Halisarca caerulea.*

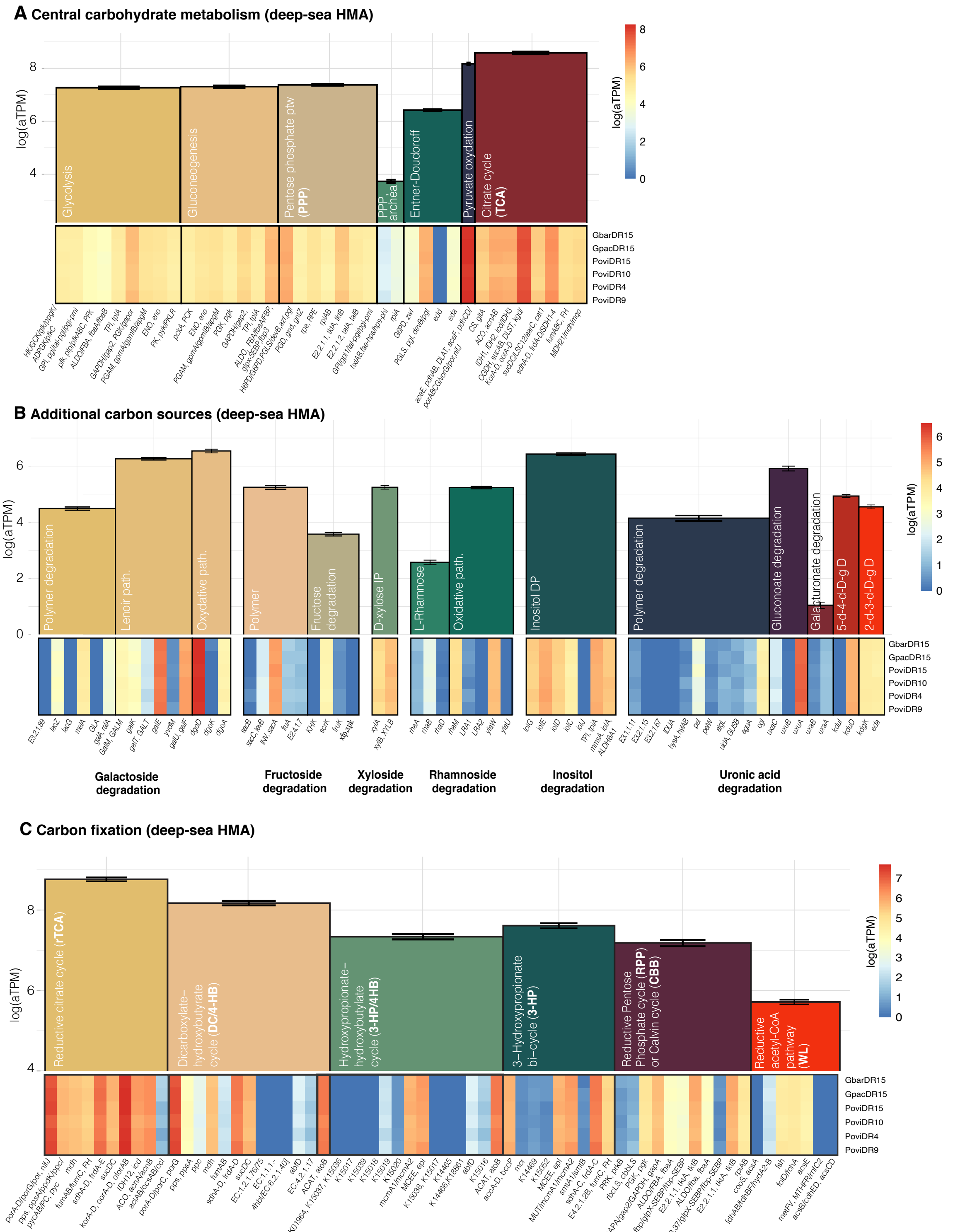

Fig. S5. Averaged gene expression values (aTMP) of KEGG modules within central carbohydrate metabolism (**A**), additional carbon sources (**B**) and carbon fixation (**C**) across deep HMA sponge. Top bars show KEGG module expression and bottom heatmap constituent gene expression.
Gbar = *G. barretti*, Gpac = *G. pachydermata*, Povi = *P. ovisternata*. DR indicates sampling sites.

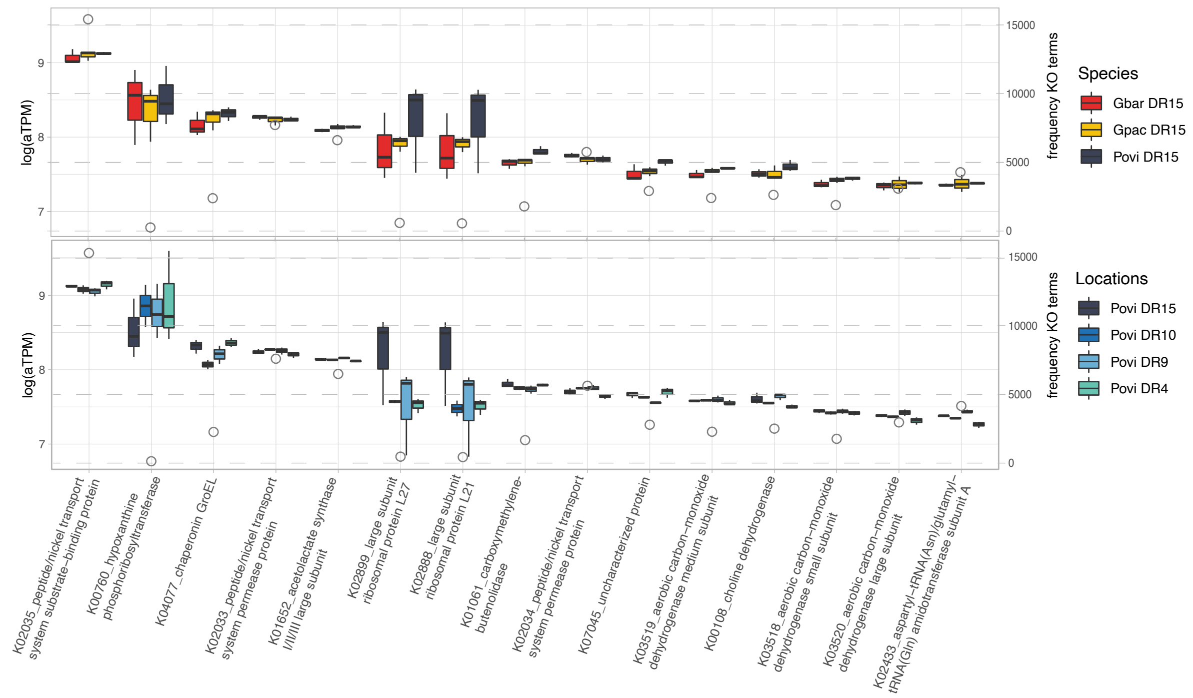

Fig. S6. The fifteen most expressed single KEGG database genes in sponge microbomes among species (**A**), and among locations. Boxplots show averaged expression values and circles show the frequency of each gene in our datasets of deep-sea HMA sponges.
Gbar = *G. barretti*, Gpac = *G. pachydermata*, Povi = *P. ovisternata*. DR indicates sampling sites.

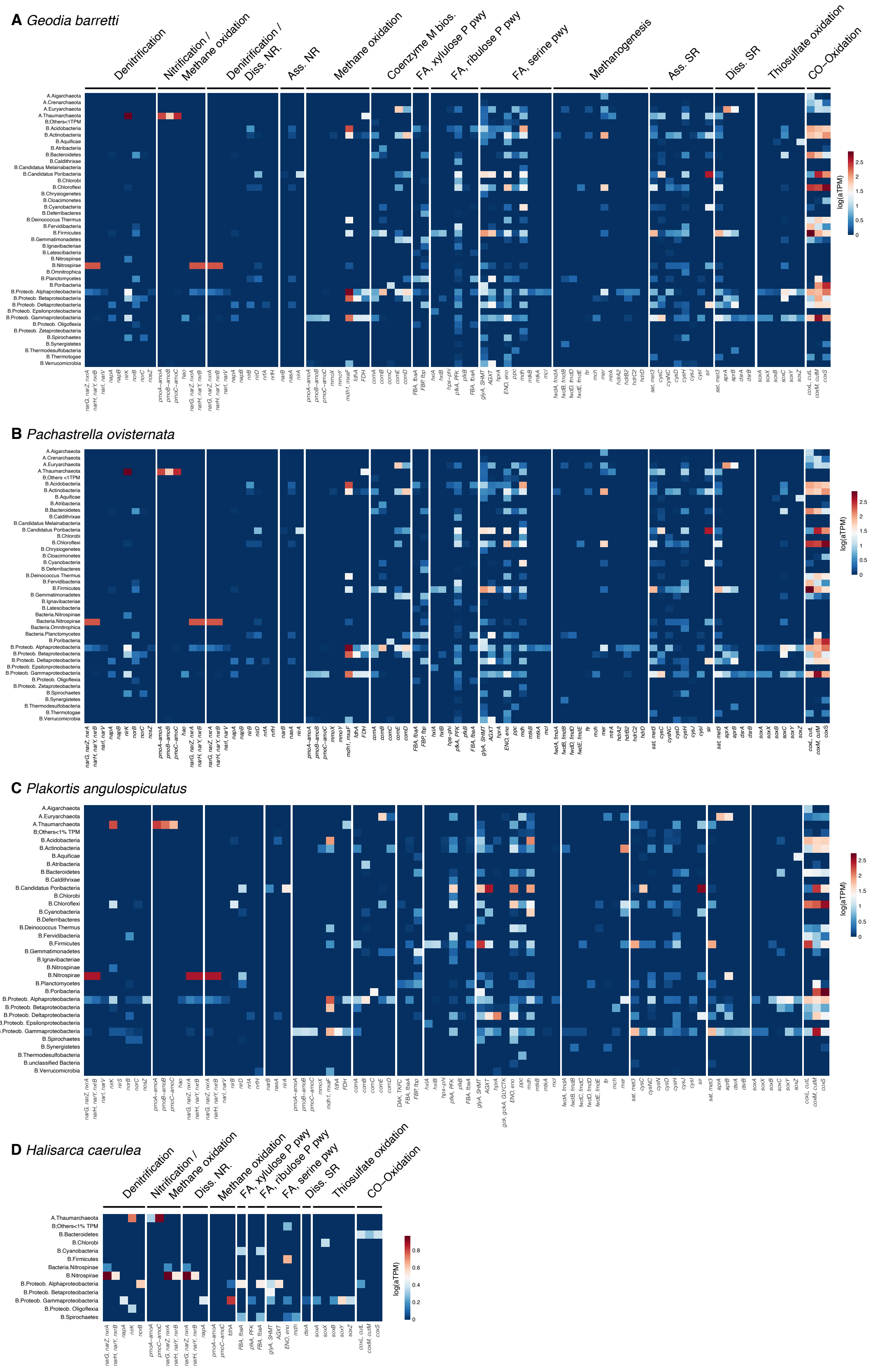

Fig. S7. Averaged sponge microbiome gene expression values (aTMP), split by microbial phyla, involved in energy metabolisms for the sponges *Geodia barretti* (**A**), *Pachastrella ovisternata* (**B**), *Plakortis angulospiculatus* (**C**) and *Halisarca caerulea* (**D**). B = Bacteria, A = Archaea. Diss. NR = Dissimilatory nitrate reduction, Ass. NR = Assimilatory nitrate reduction, Ass. SR = Assimilatory sulfate reduction, Diss. SR = Dissimilatory sulfate reduction.

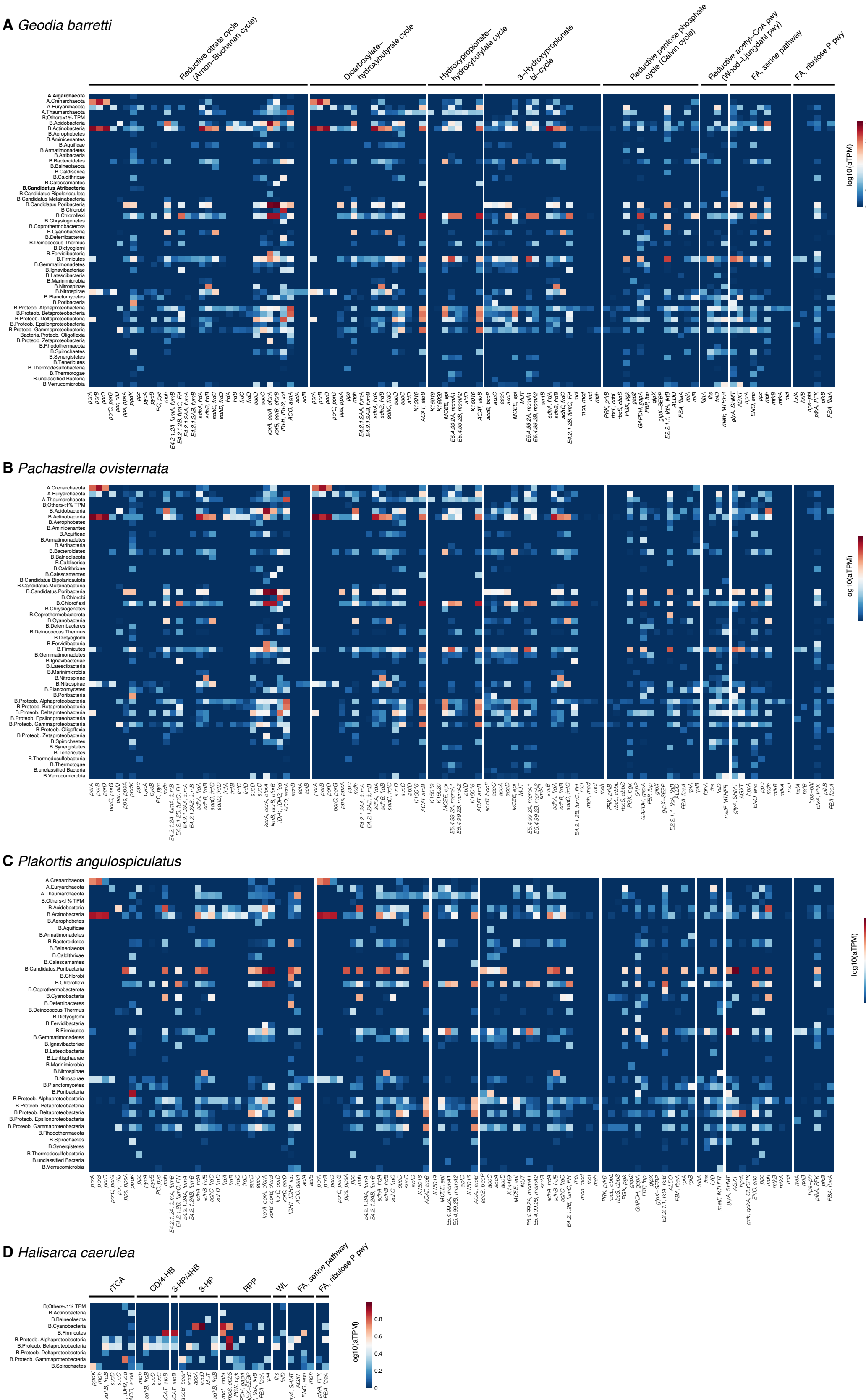

Fig. S8. Averaged sponge microbiome gene expression values, split by microbial phyla, involved in carbon fixation for the sponges *Geodia barretti* (**A**), *Pachastrella ovisternata* (**B**), *Plakortis angulospiculatus* (**C**) and *Halisarca caerulea* (**D**). B = Bacteria, A = Archaea
