# Supplementary material for: High compositional and functional similarity in the microbiome of deep-sea sponges
Source: ISME J. 2024 Jan 12;18(1):wrad030. doi: 10.1093/ismejo/wrad030 (PMC10837836; doi:10.1093/ismejo/wrad030)
Supplement: RNA_Supplementary_Text_reviewed_rev_clean_wrad030 [file rna_supplementary_text_reviewed_rev_clean_wrad030.pdf]

SUPPLEMENTARY INFORMATION

High compositional and functional similarity in the microbiome of deep-sea sponges

Cristina Díez-Vives[1,3], Ana Riesgo[2,3]

[1] Department of Systems Biology, Centro Nacional de Biotecnología, c/ Darwin, 3, 28049, Madrid, Spain

[2] Department of Biodiversity and Evolutionary Biology, Museo Nacional de Ciencias Naturales (CSIC), c/José Gutiérrez Abascal 2, 28006, Madrid, Spain

[3] Department of Life Sciences, The Natural History Museum, London, SW7 5BD, UK

Table of Contents

1 SHARED AND DIFFERENTIAL FEATURES OF DEEP-SEA SPONGE MICROBIOMES 2
1.1 LOW COMPOSITIONAL DIVERGENCE 2
1.2 LOW FUNCIONAL DIVERGENCE 2
2 GENERAL METABOLISM OF THE DEEP-SEA SPONGE MICROBIOME 3
2.1 METABOLISMS OF COFACTORS AND VITAMINS 3
2.2 LIPID METABOLISM 4
2.3 AMINO ACID METABOLISM 4
2.4 CARBOHYDRATE METABOLISM 4
2.5 ENERGY METABOLISM 5
2.5.1 Carbon fixation 5
2.5.2 Nitrogen metabolism 5
2.5.3 Methane metabolism 7
2.5.4 Sulphur metabolism 8
2.5.5 Carbon monoxide oxidation 8
2.5.6 Phosphonate utilization 9
3 OTHER FUNCTIONS RELATED TO THE SYMBIOTIC LIFESTYLE 9
4 MOST ABUNDANT INDIVIDUAL KO TERMS IN DEEP-SEA SPONGES 11
5 FUNCTIONAL CONVERGENCE 12
6 REFERENCES 14

1 SHARED AND DIFFERENTIAL FEATURES OF DEEP-SEA SPONGE MICROBIOMES

1.1 LOW COMPOSITIONAL DIVERGENCE

To further investigate similarities in the microbiome between species or locations, we analyzed the microbial members and functions that were shared between the sample groups (the aggregation of the 3 replicates from the same species and location), and the ones that differed.

Individual sponges shared in pairs between 79 to 63% of their abundant ASVs (i.e., >0.01 % RA, Fig. 3). The two most similar sample groups were Povi_DR15 and Povi_DR9 (79% shared ASVs). Interestingly Gpac_DR15 shared about 72% of ASVs with Povi_DR15, which is as similar as any two sample groups of Povi from different locations. Gbar_DR15-Gpac_DR15 shared 68%, and Gbar_DR15-Povi_DR15 shared 67%. All samples together had 44% of ASVs in common.

We then investigated what differentiated our samples by checking the differentially abundant (DA) ASVs between species and locations. As expected, few ASVs were DA between the species: 9 DA-ASV between Gpac_DR15-Povi_DR15, 14 between Gbar_DR15-Gpac_DR15, and 23 between Gbar_DR15-Povi_DR15 (out of 9,751 ASV considered >0.01% RA and >= 2 samples, Table S10, Fig. S1A). Most of the DA ASVs represented low abundant taxa, covering several genera with no clear group being representative of any species. Only one DA-ASVs assigned to Woesearchaeia (Nanoarchaeota) reached up to 2.7% of aRA and was more abundant in one Gpac sample compared to the other species. Among locations there were a maximum of three DA-ASVs detected (Table S10, Fig. S1B), and the most abundant one was an ASV assigned to Nitrososphaeria (Thaumarchaeota).

1.2 LOW FUNCIONAL DIVERGENCE

In terms of functions, pair comparisons shared between 96 to 87% of their abundant transcripts (>1 tpm, Fig. 3). Here as before, Povi_DR15 and Povi_DR9 shared the greatest number of transcripts (93%), and Gpac_DR15 shared with Povi_DR15 up to 92% of transcripts, but Gbar_DR15 and Gpac_DR15 shared 90%. In this case, locations shared more transcripts among them than among species. All Povi samples shared 89% of transcripts, and the 3 species from DR15 shared 85% of transcripts.

We also checked differentially expressed (DE) transcripts. We selected transcripts with more than 10 cpm and in more than 2 samples, leaving pairwise datasets of 18,000 – 19,000 transcripts. Relatively small differences in gene expression were also found among species, with Gpac-Povi showing the smallest differences (240 DE-trans), 686 DE-transcripts between Gbar-Gpac, and 1,132 DE-transcripts between Gbar-Povi (Table S11), this represented from 1.2 to 6% of the transcripts. These DE-transcripts included one or more enzymes that were related with a number of different pathways (Table S11), although around 70% did not have any KO annotation. From the annotated ones, the Gbar-Povi comparison showed that amino acid metabolism and biosynthesis of secondary metabolites were differentially upregulated in Povi (Fig. S2A). Quorum sensing pathways included 26 DE genes upregulated in Gbar and 51 DE genes in Povi, similar to ABC transporters (23 and 49, respectively).

Nitrogen metabolism had 19 genes upregulated in Povi, and 2 in Gbar, while Sulfur metabolism had 7 genes upregulated in Gbar. Xenobiotics biodegradation and metabolisms was notably upregulated in Povi, as well as cell adhesion molecules and ECM-receptor interaction (within signaling molecules). The comparison between Gbar-Gpac pointed to similar pathways being DE expressed, albeit to a smaller extent. And the lower 240 DE-transcripts detected between Gpac-Povi were centered notable in sensing and interacting with environment and other cells such as the quorum sensing (10 DE-genes upregulated in Povi, and 1 in Gpac), ABC transporters (9 and 8), notch signaling, two component system, and ECM-receptors. Only few genes were related with differences in nitrogen metabolism, oxidative phosphorylation and porphyrin and chlorophyll metabolism.

DE-transcripts between locations for Povi were lower, ranging from 11 DE-trans (Povi_DR9 vs Povi_DR15) to 123 DE-transcripts (Povi_DR10 vs. Povi_DR15), this is 0.05 to 0.6% of the transcripts considered (Table S11). In the latter, Povi_DR10 always presented upregulated genes in many different pathways (Fig. S2B), being noteworthy porphyrin and chlorophyl metabolism, phosphonate and phosphinate metabolism, lipid and amino acid metabolism. However, when Povi_DR10 was compared to Povi_DR4 upregulated genes were mostly associated with pathways of carbohydrate, energy, and cofactors and vitamins metabolisms (Fig. S2B), while Povi_DR4 had genes upregulated in many other functions like prodigiosin biosynthesis, quorum sensing, or lipid metabolism. The comparison Povi_DR10-Povi_DR9 featured 5 pathways notably upregulated in Povi_DR9 and none in Povi_DR10. Between Povi_DR4-Povi_DR9 (114 DE-transcripts), genes were usually upregulated in Povi_DR9 covering many different pathways, the largest differences were in butanoate metabolism, carbon fixation, and benzoate degradation among others.

Generally, these results show the relatively small differences (i.e., low percentage of differentially expressed transcripts between groups) in the microbiome of different sponge species at functional level. Any differences were centered in microbial genes related with the sensing of and exchange with their environment, and few metabolic pathways. Interestingly, *P. ovisternata* showed greater upregulation in a variety of metabolic pathways, many of them among xenobiotic biodegradation. Unfortunately, poor taxonomic classification of the associated ASV sequences at the species level did not allow us to confidently identify possible xenobiotic-degrading microorganisms.

2 GENERAL METABOLISM OF THE DEEP-SEA SPONGE MICROBIOME

2.1 METABOLISMS OF COFACTORS AND VITAMINS

Cofactors and vitamins were expressed up to an accumulated value of 28,134 averaged TPM (aTPM, Table S13, Fig. 5). The most expressed pathway was pantothenate and CoA biosynthesis (7,158 aTPM), followed by porphyrin metabolism (4,195 aTPM), folate biosynthesis (2,386 aTPM), thiamine metabolism (vitamine B1; 2,153 aTPM), biotin metabolism (vitamin B7; 2,050 aTPM), nicotinate and nicotinamide metabolism (vitamin B3; 1,893 aTPM) (Table S17). Specific modules within these pathways were high expressed: heme biosynthesis (complete, 1,839 aTPM), siroheme biosynthesis (complete, 1,923 aTPM), pimeloyl-ACP biosynthesis (complete 1,721 aTPM), thiamine biosynthesis (incomplete, 1,481 aTPM), riboflavin biosynthesis (vitB12; incomplete, 1,325 aTPM) and pantothenate

biosynthesis (complete, 1,321 aTPM) (Table S14). Genes which expression were not detected included *thi*F (K03184, EC:2.7.7.73) and *ten*I (K10810, ED: 5.3.99.10) of the synthesis of thiamine, and gene PYRP2 (K22912, EC:3.1.3.104) of the riboflavin biosynthesis. These same genes were not recovered in the microbial genomes reconstructed from *Aplysina aerophoba* [3]. Sponge symbionts are commonly enriched in genes related to the synthesis of vitamins and cofactors suggesting the symbionts may satisfy the host's demand for these essential compounds [3–10], and their high expression corroborates this idea.

2.2 LIPID METABOLISM

Fatty acids biosynthesis pathway accounted for 2,834 aTPM (Table S17), and modules inside were complete (Table S14). Sponges are rich in short chain fatty acids (FA), mid-chain branched fatty acids (MBFAs) and the sponge specific demospongic acids (long chain acids, LCFAs). Sponges seem to rely on short-chain fatty acids from bacterial origin as precursors for demospongic acids, since they have incomplete biosynthesis pathways for short-chain fatty acids [11, 12]. Genes involved in the short-chain fatty acid biosynthesis (type II FAS, *Fab*DHFGILZ) were high expressed in our metatranscritpomes. MBFAs are often abundant compounds in HMA sponges, produced by bacterial-type polyketides synthetases (PKS), and *Poribacteria* was proposed as the potential producer of these compounds [13]. In *Geodia barretti,* and other North-Atlantic deep-sea demosponges, methyl-branched fatty acids were the dominant bacterial FAs, and were used as precursors for branched LCFAs [14]. LCFAs constitute a major part of sponge membrane phospholipids (PLs) and probably serve a structural and functional role [15].

2.3 AMINO ACID METABOLISM

Modules leading to amino acid biosynthesis were mostly complete, and highly expressed (Table S14). Metagenomic studies showed the genetic potential of different SAGs to synthesize different amino acids [16]. The collective sponge microbial community seems to be able to synthetize most of amino acids, with highest expression in pathways such as valine, leucine and isoleucine biosynthesis (6,264 aTPM), phenylalanine, tyrosine and tryptophan (2,756 aTPM), lysine (2,711 aTPM), betaine (1,936 aTPM) and threonine (1,362 aTPM). Catabolism of amino acids was also highly expressed i.e., valine, leucine and isoleucine degradation (4,462 aTPM), leucine degradation (1,719 aTPM), lysine degradation (154 aTPM). Amino acids are degenerated to an α-keto-acid intermediate (pyruvate, acetyl-CoA, acetoacetyl-CoA, α-ketogluterate, succinyl-CoA, fumarate, and oxalacetate), which could enter the TCA cycle. As with microbial-derived vitamins, sponge hosts may also use bacterial derived amino acids. For example, biosynthetic pathways of several amino acids were present in the prokaryotic metatranscritpome of *Xentospongia mutua*, but host-derived transcripts only included catabolic reactions for these amino acids [6].

2.4 CARBOHYDRATE METABOLISM

Central carbohydrate metabolism included highly expressed genes within citrate cycle (TCA cycle; 5,289 aTPM), pyruvate oxidation (3,469 aTPM), pentose phosphate pathway (PPP; 1,573 aTPM), glycolysis (1,420 aTPM), and the respiratory chain as energy-producing pathways (oxidative

phosphorylation, 6,966 aTPM; Table S14). These top expressed functions suggest a large community of aerobic and heterotrophic bacteria. All these pathways included the complete expression of associated genes, except the PPP that lacked the gene encoding for enzyme phosphogluconate dehydratase (EC: 4.2.1.12). This gene was also missing in 18 symbiotic genomes of *Aplysina aerophoba* [3].

Apart from glucose, symbionts seem to be able to use a variety of additional carbon sources, based on the expression of degradation pathways for galactoside (1,297 aTPM), fructoside (225 aTPM), xyloside (213 aTPM), rhamnoside (199 aTPM), inositol (617 aTPM) or uronic acids (885 aTPM; pathway descriptions are based in key enzymes described in [17]). Some intermediate genes were not expressed at detection levels and their absence has been identified in Poribacterial and genomes *Chloroflexi* genomes, however intermediate products could be converted by other enzymes [3, 17]. Degradation of complex carbohydrates appears to be a dominant feature in sponge symbionts [17, 18]. Symbiont ability to utilize diverse carbon sources can be of relevance for the host, as the sponge feeding on dissolved and particulate organic matter from the extensive filtration activity can be mediated by the bacterial symbionts [19–21]. Moreover, symbionts can utilize compounds from the extracellular matrix of the sponge as nutrient substrates [17, 18, 22]. With the lack of sun light for photosynthesis, the symbiotic heterotrophy can perform a more important role in these deep-sea communities.

2.5 ENERGY METABOLISM

2.5.1 Carbon fixation

With respect to autotrophic carbon fixation, the cumulative aTPM values showed that the reductive citrate cycle (also called Arnon-Buchanan cycle or reductive tricarboxylic acid) presented the genes with highest expression (6,319 aTPM), followed by dicarboxylate-hydroxybutyrate cycle (3,485 aTPM), 3-hydroxypropionate bi-cycle (2,005 aTPM), reductive pentose phosphate cycle (also called Calvin Cycle, 1,306 aTPM), hydroxypropionate-hydroxybutylate cycle (1,515 aTPM) and reductive acetyl-CoA pathway (also called Wood-Ljungdahl pathway, 300 aTPM). Except for the complete reductive citrate cycle and Calvin cycle, the other modules were incomplete, similarly as in MAGs from other sponges [3, 8]. Moreover, formaldehyde can be assimilated into cell material through the ribulose monophosphate pathway (RuMP, 96.8 aTPM), or the serine cycle (866.7 aTPM). The glycerate 2-kinase [EC:2.7.1.165] of the serine cycle was missing among the KOs, but was annotated within the COGs (COG2379). The RuMP was lowly expressed, but is recognized as a widespread prokaryotic pathway involved in formaldehyde fixation and detoxification [23].

2.5.2 Nitrogen metabolism

All modules associated with nitrogen metabolism were complete: denitrification (1,176 aTPM), nitrification (1,130 aTPM), dissimilatory nitrate reduction of ammonium (DRNA, 443 aTPM) and assimilatory nitrate reduction (15 aTPM) (Table S14, Fig. 6). During nitrification, ammonia is oxidized to nitrite by the sequential actions of ammonia monooxygenase and hydroxylamine dehydrogenase, providing reductant for the cell [24]. Ammonium transporter (Amt family, K03320) was expressed at high rates (1,535 aTPM), being the most expressed type of transporter, indicating the great use of this molecule. The three genes conforming the ammonia monooxigenase operon (K10944-46, *pmo-*

*amo*CAB) were co-transcribed as a single transcript, with expression levels from 99 to 337 aTPM. Oxidation of the resultant hydroxylamine to nitrite by hydroxylamine dehydrogenase (K10535, *hao*) presented lower expression values of 4.7 aTPM. The last reaction of nitrification, done by the nitrite oxidoreductase alpha and beta subunits (the enzymes K00370-71, *nar*G*, nar*Z*, nxr*A and *nar*H, *nar*Y*, nxr*B) showed again high expression values (208 and 207.5 aTPM). The key enzyme codified by the ammonia monooxigenase (*amo*) gene in the nitrogen fixation pathway belongs to the same copper-dependent membrane monooxygenase family (CuMM) as the particulate methane monooxygenase (*p*MMO, K01944-46) and probably other CuMM containing members, and cannot be distinguished by homology alone [25]. Similarly, the nitrite oxidoreductase enzymes (K00370-71) are also used in the inverse reaction, nitrate reduction, present in denitrification and DNRA, confounding the actual expression values of each function.

Within demosponge microbiomes is the common presence of ammonia-oxidizing bacteria (AOB) and archaea (AOA), which perform the first step of nitrification [26–31] and with the AOA being more abundant than AOB both in marine sponges [32–35] and in the seawater [36, 37]. Class *Nitrososphaeria* of the phylum *Thaumarchaeota* encompasses all known ammonia oxidizing archaea (AOA) which represent a monophyletic lineage in the *amo*A phylogeny [38], and are the only archaea capable of oxidising ammonia in several shallow demosponge species [8, 30, 39], as well as in deep-sea sponges [33, 40]. Our archaeal CuMMO genes (methane/ammonia monooxygenase) had expression values up to 30 times higher than the bacterial counterparts (Fig. 7) and were classified within families *Nitrosopumilaceae* and *Ca.* Nitrososphaeraceae. Family *Nitrosopumilacea* was the most abundant taxa among the amplicon (21.1% aRA of ASVs), but they only represented 1.28% aTPM of transcripts. Baterial CuMMO (ammonia oxidizing bacteria, AOB) genes belonged to *Actinobacterias*, *Methylococcales* (*Gammaproteobacteria*) and *Methylacidiphilae* (*Verrucomicrobia*), although these clustered with the closely related particulate methane and butane monooxygenase in a phylogenetic tree (*p*MMO and *p*BMO; data not shown), and therefore were probably not involved in ammonia oxidation. To prevent overestimation of nitrification, only expression values from *Thaumarchaeota* were considered (Fig. 6 and 7). The next step in nitrification, hydroxylamine oxidation (*hao*) to nitrite, expressed by *Gammaproteobacteria*, probably serves in the detoxification of hydroxylamine, instead to contributing to nitrification, as previously suggested [8], since the ammonia oxidation gene was not confirmed for this group. Nitrite oxidizing bacteria (NOB) perform the second step of the nitrification, the oxidation of nitrite to nitrate. NOB representatives among our transcripts included mostly phylum *Nitrospirae* and some *Alphaproteobacteria*.

Nitrate resulting from nitrification can be recycled through denitrification. The first step of nitrate reduction is performed by the membrane-bound (*Nar*GHI, 423 aTPM) or periplasmic (*Nap*AB, 1.3 aTPM) nitrate reductases. The next step of denitrification, performed by the cytochrome cd1-dependent nitrite reductase (K00368; *nir*K, 726.9 aTPM), was the highest expressed gene within the nitrogen metabolism, while the copper-containing nitrite reductase (K15864, *nir*S) was expressed in low values (0.07 aTPM). Previous studies have shown that only enzymes responsible for the first steps of denitrification (nitrate reductase and nitrite reductase) are enriched in sponge symbionts, while enzymes for the final two steps, nitric oxide reductase (*nor*BC, K04561-K02305), and nitrous-oxide

reductase (*nosZ*, K00376) have been rarely detected, suggesting incomplete pathways, which would result in the release of nitrous oxide into the surrounding seawater, or the presence of alternative routes producing final N_2 and O_2 bridging major pathways [5, 20, 41–43]. Corroborating those studies, the last steps of denitrification were expressed in low values (9.6 and 0.82 aTPM, respectively), indicating that the cycle can be closed, but the lower expression could produce the observed accumulation of nitrous oxide.

In denitrification, nitric oxide reductase (*nor*B) was expressed by *Bacteroidetes*, *Deltraproteobacteria*, *Planctomycetes* and *Firmicutes*, while *nor*C was expressed by *Betaproteobacteria* and *Spirochaetes*. The last enzyme of the denitrification (*nos*Z) was expressed by *Alphaproteobacteria* and *Chloroflexi* (Fig. 7). Complete denitrification has been found in sponge-associated *Alphaproteobacteria* [44].

Inside the symbiotic cells, ammonia can be used as source of energy (nitrification) but also for biosynthesis of amino acids (ammonia assimilation). Enzymes for ammonia assimilation were also highly expressed, mostly via glutamine synthetase (K01915, *gln*A, GLUL; 732 aTPM), and in a lesser extend through glutamate dehydrogenase (K00261 GLUD1_2, *gdh*A; 55 aTPM). Ammonia can be derived from host metabolism (ammonia excretion of the sponge cells), but can also be generated by the dissimilatory nitrate reduction and by coupled denitrification and nitrogen fixation.

The enzymes characteristic of dissimilatory nitrate reduction to ammonium, DNRA (cytochrome c dependent nitrite reductases (*nrf*AH, K03385-K15876), and NADH dependent nitritre reductase (*nir*BD, K00362-63) are specific to the reduction of nitrite to ammonia and were expressed in low values (0.1 to 11 aTPM). Enzymes for the assimilatory nitrate reduction were also detected but with low expression (max. value of the ferredoxin-nitrite reductase, *nir*A, K00366, was 13 aTPM). Enzymes for the anaerobic anammox or the nitrogen fixation (NF) were not detected. In the metagenomes of *Vazella portalessi* some of the enzymes for the nitrogen pathways were missing but alternative routes were proposed to link the processes [43].

In addition, urease activity (by detection of *ure*C gene) has been identified in the sponge *Xestospongia testudinaria* [45], and urease-encoding gene clusters and urea transporters are also reported from sponge metagenomes and metatranscriptomes [46–48]. We detected expression of the three urease subunits (*ure*ABC, with accumulated 45.9 aTPM), several accessory proteinc (*ure*D-J, accumulated 53.2 aTPM), and the urea transport system (*urt*A-E with accumulated 103.2 aTPM), proving urea utilization in our deep-sea sponges. Urease genes were expressed by 14 different phyla, with dominance of Actinobacteria, and Firmicutes. Urea is one of the dominant organic nitrogenous compounds in oligotrophic oceans [49] and likely serves as an alternative nitrogen source to ammonia, nitrate, and nitrite within the sponge holobiont.

2.5.3 Methane metabolism

In the methane metabolism (3,295 aTPM), methanotrophy presented complete methane oxidation. The oxidation of methane to methanol reached expression values of 702.8 aTPM by the *pmo/amo*CAB (also annotated in nitrification) and the *mmo*XYZBCD. Similar as before, a detailed analysis indicated that only 13 *pmo*A genes belonged to the family *Methylococcaceae* (*Gammaproteobacteria*) and

Methylacidiphilum infernorum (*Verrucomicrobia*) reported as methanotrophs [50, 51]. The corrected methane-oxidizing bacteria (MOX) accounted for 40.3 aTPM of the *pmo/amo*CAB expression (Fig. 6 and 7). Methanol is further oxidized to formaldehide by the methanol dehydrogenase (cytochrome c) subunit 1 (*mdh*1*, mxa*F), expressed at 13.3 aTPM. MOX symbionts are common in animals living in deep seeps, such as worms [52], poecilosclerid sponges [53, 54] and cladorhizid sponges [55, 56].

Formaldehide can either be dissimilated to CO_2 for energy production by the formaldehyde dehydrogenase (*fdh*A, 94.3 aTPM) and formate dehydrogenase (FDH at 43.3 aTPM), or assimilated for biomass production by the xylulose monophosphate, ribulose monophosphate and serine pathways (Fig. 6). Only the ribulose monophosphate pathway was complete with an accumulated expression of 97 aTPM (Fig. S4, Table S14).

Methanogenesis was incomplete, with key enzymes such as the methyl-coenzyme M reductase (*Mcr*) complex, and methylenetetrahydromethanopterin dehydrogenase (*Mtd*) not being expressed (Fig. 6).

2.5.4 Sulphur metabolism

Sulphur is an essential nutrient for microbial synthesis of certain amino acids, vitamins and enzymes. A source of sulphur is the sponge derived taurine. Transporters for taurine acquisition (*tau*ACB, accumulated 31.9 aTPM) and degradation to sulfite (taurine dioxygensases, 54.5 aTPM) were expressed (Table S14, Fig. 7). Sulfite can be reduced to sulfide or oxidized to sulfate. Sulfate can also be produced through thiosulfate oxidation, and thiosulfate may be produced by incomplete oxidation of sulfides [8]. Modules of assimilatory sulfate reduction (ASR, 830 aTPM), dissimilatory sulfate reduction (DSR, 311 aTPM) and thiosulfate oxidation by SOX system (149 aTPM) were complete (Table S14, Fig. 7), suggesting that these processes are important in the sponge microbiome.

Both sulfate-reducing and sulfur-oxidizing microorganisms inhabit sponges, with sulfur-oxidizing bacteria (SOB) thought to oxidize the reduced sulfur compounds generated by sulfate-reducing bacteria (SRB). *Geodia barretti* showed exceptionally high sulfate reduction rates [20, 34]. SOB activities combined with chemical reoxidation processes were thought to prevent a toxic build-up of sulphide. In our *G. barretti* metatranscriptome, many different phyla were involved in these processes (Fig. 7), with a notable expression of sulfite reductase (ferredoxin) [EC:1.8.7.1] (K00392, *sir*) by *Candidatus* Poribacteria.

2.5.5 Carbon monoxide oxidation

Another potential energy source is the CO oxidation via carbon monoxide dehydrogenase (CODH)-mediated pathway. CO-oxidizing bacteria are lithoheterotrophs common in sponge microbiomes [57, 58], highly overrepresented compared to seawater [4]. The presence of this CODH complex in 65% of the sponge symbionts analysed by Burgsdorf *et al.* [59] suggested that CO oxidation is the most abundant process related to a litoheterothrophic lifestyle in sponge symbionts. Our results support this observation since CODH subunits are expressed by many different phyla at very high values in our metatranscriptomes (*cox*S at 1,668 aTPM, *cox*M at 1,922 aTPM, and *cox*L at 1,583 aTPM (Table S14, Fig. 6). The function of *cox*L is variable, and it has been related to the oxidation of CO but also oxidation

of different organic substrates in sponge symbionts [59]. CO dehydrogenase was expressed by many microbial phyla (Fig. 7), most of them were previously confirmed in other sponge species [58, 59].

2.5.6 Phosphonate utilization

Microorganisms can utilize phosphonates (reduced organiphosphorous compounds) as potential source for growth in conditions of limiting inorganic phosphorous (Pi) concentrations. Microorganisms can metabolize these compounds via cleavage of the C-P bond through at least three mechanisms: hydrolytic (2AEP, 2-aminoethylphosphate degradation pathway via *Phn*WX), radical (C-P lyase), and oxidative (*Phn*YZ) [60]. Genes for bacterial phosphate management are included in the so-called Phosphate regulon (Pho regulon), whose control is performed by a two-component regulatory system (TCRS). The involvement of the microbiome in Pi metabolism was investigated in several pathways. The general Phosphonate and phosphinate metabolism (ko00440) was expressed at 439.3 aTPM in our metatranscriptomes. Genes of the TCRS (*pho*R, *pho*B) were actively synthetized (13 to 43 aTPM). Gene products of the Pst system (phospahte-specific transport) encoding an ABC transporter of inorganic phosphate (*pst*SCAB-*pho*U operon) were expressed at 45 to 134 aTPM. During Pi starvation, TCRS activates the expression of proteins for the transport and use of phosphonates which are encoded in the C-P lyase complex and consist of 14 genes (*phn*CDEFGHIJKLMNOP). Three gene products constitute the ABC transporters for phosphonates (*phn*CDE) and were expressed from 25 to 73 aTPM. However, the seven gene products involved in the catalysis of degradation of phosphonates were expressed in low values (< 0.2 aTPM). Accessory protein *phn*P had an expression of 52.7 aTPM. Other enzymes of phosphonate catabolism such as phosponoacetate catabolism performed by *phn*A were expressed at 92.5 aTPM, and 2-AEP (2-aminoethylphosphonate) catabolism (*phn*W,*phn*X) at 4.9 and 5.5 aTPM respectively. In a global oceanic survey, bacterial C-P lyase abundance declined in the mesopleagic zone relative to the epipelagic zone, while other phosphonate degradation pathways remained well represented [61]. The proportion of phosphonate producers in free-living bacterioplankton were reported to steadily increase with depth to nearly 30-40% at 200 m [60]. Phosphate biosynthesis in our metatranscriptome was expressed, *pep*M (K01841) at 12.8 aTPM, and *ppd* (K09459) at 260.8 aTPM. It seems that phosphate metabolism in depth is focused on the biosynthesis of phosphonates. In sponges, phosphate is important for regulatory functions within the holobiont [62], genes involved in phosphorous cycle were detected in *V. pourtalessi* microbiome [43] and SAUL genome [22]. Sponge microbiome can also produce and store polyphosphate granules, which can comprise 25 to 40% of total phosphate present in sponge tissue [63], and it is conceivable that it serves as P-storage reservoir [64]. However, Acker *et al*. [60] found that phosphonate biosynthesis and catabolism pathways were mutually exclusive, therefore pointing to other proposed functions such as incorporation of phosphonates into membrane lipids or capsular polysaccharides to protect cells against enzymatic attack or phage attachment.

3 OTHER FUNCTIONS RELATED TO THE SYMBIOTIC LIFESTYLE

ATP binding cassette (ABC) transporters pathways were highly expressed (ko02010 at 9,568 aTPM, Table S17). The diverse and abundant range of membrane transporters encoded by the sponge microbiome (12,380 aTPM, Table S16) provides mechanisms to facilitate putative metabolic exchanges

[5], and indicates the strong reliance on nutrient uptake from the sponge microhabitat. Among these transporters we found high expression for amino acids, carbohydrates, lipoproteins, metals, and lipoproteins transport (Table S12). Quorum sensing pathway, includes a large family of 282 genes for sensing and transferring compounds inside the cell, which were highly expressed (ko02024 at 21,428 aTPM, Table S17), and can be used for microbiome population control [65].

Many symbiotic microorganisms are known to interact with their host via microbial secretion systems (ko03070, combined expression of 2,767 aTPM). Most genes for secretion systems Type II (TS2) were expressed, as well as Sec-RCP and Twin arginine targeting (*Tat*) for transporting proteins across the inner membrane into the periplasm (Table S17). This system excretes effectors into the milieu and processes mediated by T2S include suppression of innate immunity, adherence to host surfaces, biofilm formation, invasion into and growth within host cells, nutrient assimilation, and alterations in host ion flux [66]. The syringe-like types III, IV and VI were also expressed in our metatranscriptomes, but few genes were not detected. These systems can transport proteins across an additional host cell membrane, delivering secreted proteins directly to the cytosol of a target cell, to eliminate microbial competitors and to translocate toxic effector proteins. Genes for the secretion systems were also identified in metagenomic analysis of *Ircinia ramosa* [8]. Robbins *et al.* [39] identified that only few lineages encoded the necessary genes to form secretion systems, suggesting that eukaryotic-like repeat proteins are unlikely to be introduced in the sponge via these traditional pathways.

Eukaryotic-like repeat containing proteins (ELPs), or proteins carrying eukaryotic domains in prokaryotes, has received increasing attention for their potential involvement in mediating host–microbiome interactions [4, 5, 67–71]. ELPs from sponge-associated microbes have been shown confer ability to evade of phagocytosis [72–74]. These proteins were found to be highly enriched by sponge symbionts [4, 39, 67, 75] and were expressed across many different taxa [71]. A total of 23,394 peptides were annotated as ELPs among our peptides. TPR was the most expressed protein (3,138 aTPM), follow by WD40, Ankyrin and LRR (1,488, 1,208 and 375 aTPM, respectively). ELPs were present in 49 different phyla, but some were more prevalent in specific lineages (Table S19). For example, sponge-associated *Candidatus* Poribacteria and *Cyanobacteria* expressed larger proportions of TPR and WD40 than the others, *Proteobacteria* expressed more TPRs, *Acidobacteria* was abundant in ankyrin and TPR, *Firmicutes* in WD40 and LRR, and W40 dominated in *Cholorflexi* and *Latescibacteria*. In agreement with the results from Robbins *et al.* [39], Thaumarchaeta presented substantially lower frequency of ELPs, with what the authors explained as that the microorganism could be utilizing alternative mechanisms to maintain associations with the host.

In addition to these proteins, cell-cell adhesion molecules may be required for the attachment to the host tissue. Cadherin domains and Fibronectin III domains mediate cell adhesion and biofilm formation in eukaryotes and have also been found to serve the same function in bacteria [76–78]. These genes were enriched in the sponge-associated MAGs and identified in most bacterial lineages [39]. Genes containing cadherins and fibronectins were also high expressed in our metatranscriptomes (1,426 and 799 aTPM respectively), and taxonomically widespread to 30 phyla (Table S19). Most frequent type were cadherins expressed by *Proteobacteria*, followed by fibronectin in *Actinobaceria* and

Thaumarchaeota. In general, the high expression and wide distribution of ELPs and adhesion proteins in the sponge-associated members suggests that these features are critical for successful establishment in the sponge niche.

Production of biologically active secondary metabolites is an important defence mechanism utilised by sponges for protection against predators or epibionts [79]. Many secondary metabolites are produced by polyketide synthases (PKS), mainly Type I PKS, and non-ribosomal peptide synthetases (NRPS) [80, 81]. Some of these compounds are produced by the sponge and others by associated microorganisms [9, 82–85]. Despite being found in metagenome bins of sponge symbionts [22, 86, 87], their expression was low in our metatranscriptome. We found a total of 85 genes annotated as COG3321 (Acyl transferase domain in polyketide synthase enzymes) or COG3319 (Thioesterase domain of type I polyketide synthase) with expression of 9.6 aTPM.

Mobile genetic elements such as transposons, plasmids and prophages can facilitate adaptation to either specific niches or to changes in environmental conditions in sponge-associated microbes [5, 88]. Transposable insertion elements (selected by the category X of the COG classification: mobilome, prophages, transposons) were detected in 22,635 genes and their accumulated expression was 7,567 aTPM, across 53 different phyla, dominating *Proteobacteria* and *Cyanobacteria* (Table S20). Highest expression values were detected in transposases (COG0675, COG3293, COG3415) and a retron-type reverse transcriptase (COG3344/PF00078) previously detected in members of the 'sponge-associated unclassified lineage' (SAUL) that are frequently recorded from sponges [22].

Restriction-modification systems (R-M) are considered bacterial defence systems against incoming, foreign DNA, that are also involved in adaptation to changes in the environmental conditions, and in host colonization time protect against DNA exchange with non-symbiont and/or pathogen microorganisms [89–92]. R-M systems have been described in sponge symbionts [18, 22, 93] and may facilitate horizontal DNA exchange between sponge symbionts. These elements were annotated in 10,865 transcripts with 1,806 aTPM (highest COG0863, DNA modification methylase) (Table S21).

Members of sponge microbial communities have also incorporated into their genomes systems to effectively protect themselves and minimise the introduction of foreign DNA into their chromosomes [5, 89]. In this context, clustered regularly interspaced short palindromic repeats (CRISPRs) and their associated proteins (Cas) [94] are commonly enriched in sponge-associated microbial communities compared to seawater [4, 5, 22, 75, 89, 95, 96], with only one gene, a CRISP-nuclease (COG3513), present both in sponges and seawater [89]. The expression of genes annotated as CRISPR and their associated proteins (*Cas*) were not particularly high expressed in our metatranscriptome (the accumulated expression of 3,728 CRISPR-related transcripts accounted for 361 aTPM, Table S22), suggesting low pressure from potential invading DNA in microbes living within deep-sea sponges.

4 MOST ABUNDANT INDIVIDUAL KO TERMS IN DEEP-SEA SPONGES

Among the fifteen most abundant individual terms (KO) for all species (Fig. S6), we found three proteins related with a peptide/nickel system: a substrate binding protein (K02035 at 8,934 aTPM), and two permease proteins (K02033 and K02034, with 3,698 aTPM and 2,243 aTPM respectively). Proteins

for the transport of peptides/nickel were also the most abundant genes in the metagenomes of several tropical *Ircinia* spp. [97] and in a metaproteomic study of *Aplysina aerophoba*. These were followed by proteins for the transport of sugars (K02027) and amino acids (K01999) [98], that were also among our top 30 most expressed genes (Table S12). The metal nickel is a fundamental cofactor of many enzymatic reactions of prokaryotes and eukaryotes. Nickel-containing enzymes are involved in at least five metabolic processes, including the production and consumption of molecular hydrogen, hydrolysis of urea, reversible oxidation of carbon monoxide under anoxic conditions, methanogenesis, and detoxification of superoxide anion radicals [99]. The peptide/nickel transporter system has been hypothesized as a response to the presence of antibiotic chemicals, conferring resistance to vancomycin [100].

Hypoxanthine phosphoribosyltransferase (K00760, 6,160aTPM), the second most expressed protein in this study, is involved in the purine salvage pathway (nucleotide metabolism), catalysing the conversion of hypoxanthine and guanine to their respective mononucleotides and which is essential for life processes. The chaperonin GroEL (K04077, 3,810 aTPM), also referred as heat-shock protein family 60, and other molecular chaperons help to compensate protein stability due to heat stress [101] or other stressors such as salt or ethanol stress, osmotic pressure, presence of reactive oxygen species and toxic compounds [102, 103]. However, expression in obligate symbionts is usually high even under nonstress conditions (i.e., constitutive) [104], being commonly the most highly expressed protein in symbionts of insects [105–108]. GroEL has being proposed for several roles in microorganism-insect interaction [104, 109, 110]. For instance, in primary symbionts with reduced genomes, the chaperonin could assist in the folding of conformationally damaged proteins created by the negative effects of deleterious mutations occurring due to genome erosion [109, 111], but also as target of antimicrobial peptides (AMPs) for endosymbiont control, limiting cell division of endosymbionts [110]. High abundance or expression of GroEL genes have been reported for several sponges [67, 98, 112], although its function within sponges is not completely understood. Ribosomal proteins (L27, K02899, and L21, K02888) help in stabilizing the specific structures of rRNAs in mature subunits and facilitating the accurate folding of rRNAs during ribosome assembly [113]. These proteins perform a fundamental role in cell physiology, and therefore their high abundance was not surprising (2,612 aTPM and 2,574 aTPM, respectively).

5 FUNCTIONAL CONVERGENCE

We investigated the functional convergence of the sponge microbiome in our target species by focusing on the community members preforming the same metabolic functions in the metatranscriptome. Each annotated gene (KO) was expressed by up to 38 different phyla (Table S12) and half of them were common to more than 12 phyla. For example, nitric oxide reductase (*nor*B) was expressed by *Planctomycetes* (2.9 aTPM), *Bacteroidetes* (2.8 aTPM), *Deltraproteobacteria* (1.12 aTPM), and *Betaproteobacteria* (1 aTPM). *Nor*C subunit was expressed by *Betaproteobacteria* (0.43 aTPM) and *Spirochaetes* (0.34 aTPM). The last enzyme of the denitrification (*nos*Z) was detected in *Alphaproteobacteria* (0.80 aTPM) and *Chloroflexi* (0.12 aTPM) (Fig. 7A). The adenosine-5′-phosphosulfate reductase alpha subunit (*apr*A), a key enzyme in microbial sulfate reduction and sulfur

oxidation, was expressed mostly by *Euryarchaeota*, but also by *Alpha*-, *Beta*-, *Delta*- and *Gammaproteobacteria*, and *Fervidibacteria* (Fig. 7A). Another metabolic module with high functional redundancy was CO-oxidation, with many bacterial and archaeal taxa showing high expression values of the carbon monoxide dehydrogenase (CODH) genes *cox*L, *cox*M, and *cox*S (Fig 7A). Several aerobic bacteria genera were reported previously to utilize CO as energy supplement when organic substrates are limiting [58, 114, 115]. Carbon fixation reactions showed wide distribution among many microbial phyla (Fig 7B).

The shallow-water HMA sponge, *P. angulospiculatus*, presented similar expression of microbial taxa for the described metabolic reactions (Fig. S7C). Some differences were identified among formaldehyde assimilation modules, where Poribacteria, Firmicutes, and Deltaproteobacteria expressed several genes (e. g., AGTX, ENO, *mdh*, *pfk*A, *hpr*A, and *gly*A,) at higher rates than our deep-sea sponges. These modules had a larger percentage of completion in this shallow HMA sponge than in the deep-sea HMA sponges (Fig. S4). In carbon fixation modules, *Poribacteria* also had a higher participation in several reactions than in the deep-sea water species (Fig. S8C). In *H. caerulea*, there were many reactions and enzymes absent, but among the ones present, the main microbial taxa were shared with the HMA species, except for *Poribacteria* (Fig. S7D and S8D).

Functional redundancy has also been identified among these processes as the use of different enzymes performing equivalent reactions. In denitrification and ammonia oxidation, Fan *et al.* [5] noticed a preference of copper-containing *nir*S genes in the symbiotic community of *Cymbastella concentrica*, while cytochrome cd-1 dependent *nir*K was more frequent in other species performing the same reaction. In our deep-sea sponges, *nir*S was expressed at low values (from 0 to 0.2 tpm), but *nir*K was highly expressed (from 573 to 950 tpm), even though previous work could only amplify *nir*S in *G. barretti* [34]. These values were equivalent in all three species. We also found a dominance of membrane-bound *nar*G (208 aTPM) expression compared to periplasmic *nap*A (1.3 aTPM) in all our species (Fig 7A), contrary to earlier reports of different frequencies of these genes in different sponge species [5, 95].

There is extensive functional convergence of the microbiome in sponges [5, 30], and therefore, sponge microbiomes may share a set of core functional genes rather than a common set of taxa [116–118]. In effect, this is what was termed “guilds” [119], a group of species that exploit the same class of environmental resources in a similar way, regardless of taxonomic position. A guild with many members will contribute to the stability of the ecosystem [120, 121], because as environmental conditions shift, different members of the guild will become dominant, but the function will be carried out anyway. However, members of a guild are supposed to interact mostly by competing with each other, since they use the same resources in a similar way [122]. It is likely that the remarkable similarity of the microbiome across deep-sea sponge species, prevented us from detecting variations in the expression of different genes between the sponge species, but the number of members expressing the same gene within a sponge indicates that the sponge environment is a stable ecosystem with large functional redundancy.

6 REFERENCES

1. Li B, Dewey CN. RSEM: accurate transcript quantification from RNA-Seq data with or without a reference genome. *BMC Bioinformatics* 2011; **12**: 323.
2. Markowitz VM, Chen IMA, Palaniappan K, Chu K, Szeto E, Pillay M, *et al*. IMG 4 version of the integrated microbial genomes comparative analysis system. *Nucleic Acids Res* 2014; **42**(D1): D560-D567
3. Bayer K, Jahn MT, Slaby BM, Moitinho-Silva L, Hentschel U. Marine sponges as *Chloroflexi* hot spots: Genomic Insights and High-Resolution Visualization of an Abundant and Diverse Symbiotic Clade. *mSystems* 2018; **3**(6), 10-1128.
4. Thomas T, Rusch D, DeMaere MZ, Yung PY, Lewis M, Halpern A, *et al*. Functional genomic signatures of sponge bacteria reveal unique and shared features of symbiosis. *ISME J* 2010; **4**: 1557–1567.
5. Fan L, Reynolds D, Liu M, Stark M, Kjelleberg S, Webster NS, *et al*. Functional equivalence and evolutionary convergence in complex communities of microbial sponge symbionts. *Proc Natl Acad Sci U S A* 2012; **109**(27): E1878-E1887.
6. Fiore CL, Labrie M, Jarett JK, Lesser MP. Transcriptional activity of the giant barrel sponge, *Xestospongia muta* Holobiont: Molecular evidence for metabolic interchange. *Front Microbiol* 2015; **6**: 364.
7. Lackner G, Peters EE, Helfrich EJN, Piel J. Insights into the lifestyle of uncultured bacterial natural product factories associated with marine sponges. *Proc Natl Acad Sci U S A* 2017; **114**: E347–E356.
8. Engelberts JP, Robbins SJ, de Goeij JM, Aranda M, Bell SC, Webster NS. Characterization of a sponge microbiome using an integrative genome-centric approach. *ISME J* 2020; **14**: 1100–1110.
9. Hentschel U, Piel J, Degnan SM, Taylor MW. Genomic insights into the marine sponge microbiome. *Nat Rev Microbiol* 2012; **10**: 641–654.
10. Radax R, Rattei T, Lanzen A, Bayer C, Rapp HT, Urich T, *et al*. Metatranscriptomics of the marine sponge *Geodia barretti*: Tackling phylogeny and function of its microbial community. *Environ Microbiol* 2012; **14**: 1308–1324.
11. Srivastava M, Simakov O, Chapman J, Fahey B, Gauthier MEA, Mitros T, *et al*. The *Amphimedon queenslandica* genome and the evolution of animal complexity. *Nature* 2010; **466**: 720–726.
12. Germer J, Cerveau N, Jackson DJ. The holo-transcriptome of a calcified early branching metazoan. *Front Mar Sci* 2017; **4**: 81.
13. Hochmuth T, Niederkrüger H, Gernert C, Siegl A, Taudien S, Platzer M, *et al*. Linking chemical and microbial diversity in marine sponges: Possible role for poribacteria as producers of methyl-branched fatty acids. *ChemBioChem* 2010; **11**: 2572–2578.
14. de Kluijver A, Nierop KGJ, Morganti TM, Bart MC, Slaby BM, Hanz U, *et al*. Bacterial precursors and unsaturated long-chain fatty acids are biomarkers of North-Atlantic deep-sea demosponges. *PLoS One* 2021; **16**(1): e0241095.
15. Lawson MP, Thompson JE, Djerassi C. Cell membrane localization of long chain C24−C30 fatty acids in two marine demosponges. *Lipids* 1988; **23**: 741–749.
16. Bayer K, Busch K, Kenchington E, Beazley L, Franzenburg S, Michels J, *et al*. Microbial Strategies for Survival in the Glass Sponge *Vazella pourtalesii*. *mSystems* 2020; **5**(4): 10-1128.
17. Kamke J, Sczyrba A, Ivanova N, Schwientek P, Rinke C, Mavromatis K, *et al*. Single-cell genomics reveals complex carbohydrate degradation patterns in poribacterial symbionts of marine sponges. *ISME J* 2013; **7**: 2287–2300.

18. Slaby BM, Hackl T, Horn H, Bayer K, Hentschel U. Metagenomic binning of a marine sponge microbiome reveals unity in defense but metabolic specialization. *ISME J* 2017; **11**: 2465–2478.
19. de Goeij JM, Moodley L, Houtekamer M, Carballeira NM, van Duyl FC. Tracing 13C-enriched dissolved and particulate organic carbon in the bacteria-containing coral reef sponge *Halisarca caerulea*: Evidence for DOM-feeding. *Limnol Oceanogr* 2008; **53**: 1376–1386.
20. Webster NS, Thomas T. The sponge hologenome. *mBio* 2016; **7**(2): 10-1128.
21. Pita L, Rix L, Slaby BM, Franke A, Hentschel U. The sponge holobiont in a changing ocean: from microbes to ecosystems. *Microbiome* 2018; **6**: 46
22. Astudillo-García C, Slaby BM, Waite DW, Bayer K, Hentschel U, Taylor MW. Phylogeny and genomics of SAUL, an enigmatic bacterial lineage frequently associated with marine sponges. *Environ Microbiol* 2018; **20**: 561–576.
23. Orita I, Sato T, Yurimoto H, Kato N, Atomi H, Imanaka T, *et al*. The ribulose monophosphate pathway substitutes for the missing pentose phosphate pathway in the archaeon *Thermococcus kodakaraensis*. *J Bacteriol* 2006; **188**: 4698–4704.
24. Wood PM. Nitrification as a bacterial energy source. *Nitrification, Oxford, Soc Gen Microbi* 1986; 39–62.
25. Arp DJ, Stein LY. Metabolism of Inorganic N Compounds by Ammonia-Oxidizing Bacteria. *Crit Rev Biochem Mol Biol* 2003; **38**: 471–495
26. Bayer K, Schmitt S, Hentschel U. Physiology, phylogeny and in situ evidence for bacterial and archaeal nitrifiers in the marine sponge *Aplysina aerophoba*. *Environ Microbiol* 2008; **10**: 2942–2955.
27. Subina NS, Thorat BR, Gonsalves MJ. Nitrification in intertidal sponge *Cinachyrella cavernosa*. *Aquat Ecol* 2018; **52**: 155–164.
28. Southwell MW, Weisz JB, Martens CS, Lindquist N. In situ fluxes of dissolved inorganic nitrogen from the sponge community on Conch Reef, Key Largo, Florida. *Limnol Oceanogr* 2008; **53**: 986–996.
29. Schläppy ML, Schöttner SI, Lavik G, Kuypers MMM, de Beer D, Hoffmann F. Evidence of nitrification and denitrification in high and low microbial abundance sponges. *Mar Biol* 2010; **157**: 593–602.
30. Ribes M, Jiménez E, Yahel G, López-Sendino P, Diez B, Massana R, *et al*. Functional convergence of microbes associated with temperate marine sponges. *Environ Microbiol* 2012; **14**: 1224–1239.
31. Morganti T, Coma R, Yahel G, Ribes M. Trophic niche separation that facilitates co-existence of high and low microbial abundance sponges is revealed by in situ study of carbon and nitrogen fluxes. *Limnol Oceanogr* 2017; **62**: 1963–1983.
32. Radax R, Hoffmann F, Rapp HT, Leininger S, Schleper C. Ammonia-oxidizing archaea as main drivers of nitrification in cold-water sponges. *Environ Microbiol* 2012; **14**: 909–923.
33. Steinert G, Busch K, Bayer K, Kodami S, Arbizu PM, Kelly M, *et al*. Compositional and quantitative insights into bacterial and archaeal communities of south Pacific deep-sea sponges (*Demospongiae* and *Hexactinellida*). *Front Microbiol* 2020; **11**: 716.
34. Hoffmann F, Radax R, Woebken D, Holtappels M, Lavik G, Rapp HT, *et al*. Complex nitrogen cycling in the sponge *Geodia barretti*. *Environ Microbiol* 2009; **11**: 2228–2243.
35. Li ZY, Wang YZ, He LM, Zheng HJ. Metabolic profiles of prokaryotic and eukaryotic communities in deep-sea sponge *Lamellomorpha* sp. indicated by metagenomics. *Sci Rep* 2014; **4**(1): 3895.

36. Wuchter C, Abbas B, Coolen MJL, Herfort L, van Bleijswijk J, Timmers P, *et al.* Archaeal nitrification in the ocean. *Proc Natl Acad Sci U S A* 2006; **103**: 12317–12322.
37. Francis CA, Roberts KJ, Beman JM, Santoro AE, Oakley BB. Ubiquity and diversity of ammonia-oxidizing archaea in water columns and sediments of the ocean. *Proc Natl Acad Sci U S A* 2005; **102**: 14683–14688.
38. Alves RJE, Minh BQ, Urich T, Von Haeseler A, Schleper C. Unifying the global phylogeny and environmental distribution of ammonia-oxidising archaea based on amoA genes. *Nat Commun* 2018; **9**(1): 1517.
39. Robbins SJ, Song W, Engelberts JP, Glasl B, Slaby BM, Boyd J, *et al.* A genomic view of the microbiome of coral reef demosponges. *ISME J* 2021; **15**: 1641–1654.
40. Tian RM, Sun J, Cai L, Zhang WP, Zhou GW, Qiu JW, *et al.* The deep-sea glass sponge *Lophophysema eversa* harbours potential symbionts responsible for the nutrient conversions of carbon, nitrogen and sulfur. *Environ Microbiol* 2016; **18**: 2481–2494.
41. Ettwig KF, Butler MK, Le Paslier D, Pelletier E, Mangenot S, Kuypers MMM, *et al.* Nitrite-driven anaerobic methane oxidation by oxygenic bacteria. *Nature* 2010; **464**: 543–548.
42. Karimi E, Slaby BM, Soares AR, Blom J, Hentschel U, Costa R. Metagenomic binning reveals versatile nutrient cycling and distinct adaptive features in alphaproteobacterial symbionts of marine sponges. *FEMS Microbiol Ecol* 2018; **94**(6): fiy074.
43. Maldonado M, López-Acosta M, Busch K, Slaby BM, Bayer K, Beazley L, *et al.* A microbial nitrogen engine modulated by bacteriosyncytia in hexactinellid sponges: Ecological implications for deep-sea communities. *Front Mar Sci* 2021; **8**: 638505.
44. Bondarev V, Richter M, Romano S, Piel J, Schwedt A, Schulz-Vogt HN. The genus Pseudovibrio contains metabolically versatile bacteria adapted for symbiosis. *Environ Microbiol* 2013; **15**: 2095–2113.
45. Su J, Jin L, Jiang Q, Sun W, Zhang F, Li Z. Phylogenetically diverse ureC genes and their expression suggest the urea utilization by bacterial symbionts in marine sponge *Xestospongia testudinaria*. *PLoS One* 2013; **8**(5): e64848.
46. Siegl A, Kamke J, Hochmuth T, Piel J, Richter M, Liang C, *et al.* Single-cell genomics reveals the lifestyle of *Poribacteria*, a candidate phylum symbiotically associated with marine sponges. *ISME J* 2011; **5**: 61–70.
47. Hallam SJ, Konstantinidis KT, Putnam N, Schleper C, Watanabe Y, Sugahara J, *et al.* Genomic analysis of the uncultivated marine crenarchaeote *Cenarchaeum symbiosum*. *Proc Natl Acad Sci U S A* 2006; **103**: 18296–18301.
48. Moitinho-Silva L, Díez-Vives C, Batani G, Esteves AIS, Jahn MT, Thomas T. Integrated metabolism in sponge-microbe symbiosis revealed by genome-centered metatranscriptomics. *ISME J* 2017; **11**: 1651–1666.
49. Bronk DA, Glibert PM, Ward BB. Nitrogen uptake, dissolved organic nitrogen release, and new production. *Science* 1994; **265**: 1843–1846.
50. Semrau JD, DiSpirito AA, Murrell JC. Life in the extreme: thermoacidophilic methanotrophy. *Trends Microbiol* 2008; **16**: 190–193
51. Murrell JC. The Aerobic Methane Oxidizing Bacteria (Methanotrophs). *Handbook of Hydrocarbon and Lipid Microbiology*. 2010. Springer Berlin Heidelberg, pp 1953–1966.
52. Goffredi SK, Tilic E, Mullin SW, Dawson KS, Keller A, Lee RW, *et al.* Methanotrophic bacterial symbionts fuel dense populations of deep-sea feather duster worms (Sabellida, Annelida) and extend the spatial influence of methane seepage. *Sci Adv* 2020; **6**(14): eaay8562.
53. Nishijima M, Lindsay DJ, Hata J, Nakamura A, Kasai H, Ise Y, *et al.* Association of thioautotrophic bacteria with deep-sea sponges. *Mar Biotechnol* 2010; **12**: 253–260.

54. Rubin-Blum M, Antony CP, Sayavedra L, Martínez-Pérez C, Birgel D, Peckmann J, *et al*. Fueled by methane: deep-sea sponges from asphalt seeps gain their nutrition from methane-oxidizing symbionts. *ISME J* 2019; **13**: 1209–1225.
55. Vacelet J, Fiala-Médioni A, Fisher C, Boury-Esnault N. Symbiosis between methane-oxidizing bacteria and a deep-sea carnivorous cladorhizid sponge. *Mar Ecol Prog Ser* 1996; **145**: 77–85.
56. Hestetun JT, Dahle H, Jørgensen SL, Olsen BR, Rapp HT. The microbiome and occurrence of methanotrophy in carnivorous sponges. *Front Microbiol* 2016; **7**: 1781.
57. Feng G, Li Z. Carbon and Nitrogen Metabolism of Sponge Microbiome. *Symbiotic Microbiomes of Coral Reefs Sponges and Corals*. 2019. Springer Netherlands, Dordrecht, pp 145–169.
58. Feng G, Zhang F, Banakar S, Karlep L, Li Z. Analysis of functional gene transcripts suggests active CO2 assimilation and CO oxidation by diverse bacteria in marine sponges. *FEMS Microbiol Ecol* 2019; **95**(7): fiz087.
59. Burgsdorf I, Sizikov S, Squatrito V, Britstein M, Slaby BM, Cerrano C, *et al*. Lineage-specific energy and carbon metabolism of sponge symbionts and contributions to the host carbon pool. *ISME J* 2022; **16**: 1163–1175.
60. Acker M, Hogle SL, Berube PM, Hackl T, Coe A, Stepanauskas R, *et al*. Phosphonate production by marine microbes: Exploring new sources and potential function. *Proc Natl Acad Sci U S A* 2022; **119**(11): e2113386119.
61. Sosa OA, Repeta DJ, DeLong EF, Ashkezari MD, Karl DM. Phosphate-limited ocean regions select for bacterial populations enriched in the carbon–phosphorus lyase pathway for phosphonate degradation. *Environ Microbiol* 2019; **21**: 2402–2414.
62. Rao NN, Gómez-García MR, Kornberg A. Inorganic polyphosphate: Essential for growth and survival. *Annu Rev Biochem* 2009; **78**: 605–647
63. Zhang F, Blasiak LC, Karolin JO, Powell RJ, Geddes CD, Hill RT, *et al*. Phosphorus sequestration in the form of polyphosphate by microbial symbionts in marine sponges. *Proc Natl Acad Sci U S A* 2015; **112**: 4381–4386.
64. Colman AS. Sponge symbionts and the marine P cycle. *Proc Natl Acad Sci U S A* 2015; **112**: 4191–4192.
65. Díez-Vives C, Koutsouveli V, Conejero M, Riesgo A. Global patterns in symbiont selection and transmission strategies in sponges. *Front Ecol Evol* 2022; **10**: 1015592.
66. Cianciotto NP, White RC. Expanding role of type II secretion in bacterial pathogenesis and beyond. *Infect Immun* 2017; **85**: 10-1128.
67. Liu M, Fan L, Zhong L, Kjelleberg S, Thomas T. Metaproteogenomic analysis of a community of sponge symbionts. *ISME J* 2012; **6**: 1515–1525.
68. Gao ZM, Wang Y, Tian RM, Wong YH, Batang ZB, Al-Suwailem AM, *et al*. Symbiotic Adaptation Drives Genome Streamlining of the Cyanobacterial Sponge Symbiont "*Candidatus* Synechococcus spongiarum". *mBio* 2014; **5**(2): 10-1128.
69. Kamke J, Rinke C, Schwientek P, Mavromatis K, Ivanova N, Sczyrba A, *et al*. The candidate phylum *Poribacteria* by single-cell genomics: New insights into phylogeny, cell-compartmentation, eukaryote-like repeat proteins, and other genomic features. *PLoS One* 2014; **9**(1): e87353.
70. Gomez-Valero L, Rusniok C, Cazalet C, Buchrieser C. Comparative and functional genomics of legionella identified eukaryotic like proteins as key players in host-pathogen interactions. *Front Microbiol* 2011; **2**: 208.
71. Díez-Vives C, Moitinho-Silva L, Nielsen S, Reynolds D, Thomas T. Expression of eukaryotic-like protein in the microbiome of sponges. *Mol Ecol* 2017; **26**: 1432–1451.
72. Nguyen MTHD, Liu M, Thomas T. Ankyrin-repeat proteins from sponge symbionts modulate amoebal phagocytosis. *Mol Ecol* 2014; **23**: 1635–1645.

73. Reynolds D, Thomas T. Evolution and function of eukaryotic-like proteins from sponge symbionts. *Mol Ecol* 2016; **25**: 5242–5253.
74. Jahn MT, Arkhipova K, Markert SM, Stigloher C, Lachnit T, Pita L, *et al.* A Phage Protein Aids Bacterial Symbionts in Eukaryote Immune Evasion. *Cell Host Microbe* 2019; **26**: 542-550.e5.
75. Burgsdorf I, Slaby BM, Handley KM, Haber M, Blom J, Marshall CW, *et al.* Lifestyle evolution in cyanobacterial symbionts of sponges. *mBio* 2015; **6**(3): 10-1128.
76. Schwarz-Linek U, Werner JM, Pickford AR, Gurusiddappa S, Ewa JHK, Pilka S, *et al.* Pathogenic bacteria attach to human fibronectin through a tandem β-zipper. *Nature* 2003; **423**: 177–181.
77. Hymes JP, Klaenhammer TR. Stuck in the middle: Fibronectin-binding proteins in gram-positive bacteria. *Front Microbiol* 2016; **7**: 1504.
78. Fraiberg M, Borovok I, Weiner RM, Lamed R. Discovery and characterization of cadherin domains in Saccharophagus degradans 2-40. *J Bacteriol* 2010; **192**: 1066–1074.
79. Pawlik JR. The Chemical Ecology of Sponges on Caribbean Reefs: Natural Products Shape Natural Systems. *Bioscience* 2011; **61**: 888–898.
80. Fischbach MA, Walsh CT. Assembly-line enzymology for polyketide and nonribosomal peptide antibiotics: Logic machinery, and mechanisms. *Chem Rev* 2006; **106**: 3468–3496.
81. Hwang S, Lee N, Cho S, Palsson B, Cho BK. Repurposing Modular Polyketide Synthases and Non-ribosomal Peptide Synthetases for Novel Chemical Biosynthesis. *Front Mol Biosci* 2020; **7**: 87.
82. Trindade-Silva AE, Rua CPJ, Andrade BGN, Vicente ACP, Silva GGZ, Berlinck RGS, *et al.* Polyketide synthase gene diversity within the microbiome of the sponge *Arenosclera brasiliensis*, endemic to the southern Atlantic Ocean. *Appl Environ Microbiol* 2013; **79**: 1598–1605.
83. Piel J. Metabolites from symbiotic bacteria. *Nat Prod Rep* 2004; **21**: 519–538
84. Wilson MC, Mori T, Rückert C, Uria AR, Helf MJ, Takada K, *et al.* An environmental bacterial taxon with a large and distinct metabolic repertoire. *Nature* 2014; **506**: 58–62.
85. Flórez L V., Biedermann PHW, Engl T, Kaltenpoth M. Defensive symbioses of animals with prokaryotic and eukaryotic microorganisms. *Nat Prod Rep* 2015; **32**: 904–936.
86. Sala G Della, Hochmuth T, Teta R, Costantino V, Mangoni A. Polyketide synthases in the microbiome of the marine sponge *plakortis halichondrioides*: A metagenomic update. *Mar Drugs* 2014; **12**: 5425–5440.
87. Fieseler L, Hentschel U, Grozdanov L, Schirmer A, Wen G, Platzer M, *et al.* Widespread occurrence and genomic context of unusually small polyketide synthase genes in microbial consortia associated with marine sponges. *Appl Environ Microbiol* 2007; **73**: 2144–2155.
88. Alex A, Antunes A. Whole genome sequencing of the symbiont *Pseudovibrio* sp. from the intertidal marine sponge *Polymastia penicillus* revealed a gene repertoire for host-switching permissive lifestyle. *Genome Biol Evol* 2015; **7**: 3022–3032.
89. Horn H, Slaby BM, Jahn MT, Bayer K, Moitinho-Silva L, Förster F, *et al.* An Enrichment of CRISPR and other defense-related features in marine sponge-associated microbial metagenomes. *Front Microbiol* 2016; **7**: 1751.
90. Zheng H, Dietrich C, Hongoh Y, Brune A. Restriction-modification systems as mobile genetic elements in the evolution of an intracellular symbiont. *Mol Biol Evol* 2016; **33**: 721–725.

91. Ershova AS, Rusinov IS, Spirin SA, Karyagina AS, Alexeevski A V. Role of restriction-modification systems in prokaryotic evolution and ecology. *Biochemistry (Moscow)* 2015; **80**: 1373–1386.
92. Vasu K, Nagaraja V. Diverse Functions of Restriction-Modification Systems in Addition to Cellular Defense. *Microbiol Mol Biol R* 2013; **77**: 53–72.
93. Gauthier MEA, Watson JR, Degnan SM. Draft genomes shed light on the dual bacterial symbiosis that dominates the microbiome of the coral reef sponge *Amphimedon queenslandica*. *Front Mar Sci* 2016; **3**: 196.
94. Makarova KS, Haft DH, Barrangou R, Brouns SJJ, Charpentier E, Horvath P, *et al*. Evolution and classification of the CRISPR-Cas systems. *Nat Rev Microbiol* 2011; **9**: 467–477.
95. Moreno-Pino M, Cristi A, Gillooly JF, Trefault N. Characterizing the microbiomes of Antarctic sponges: a functional metagenomic approach. *Sci Rep* 2020; **10**(1): 645.
96. Podell S, Blanton JM, Oliver A, Schorn MA, Agarwal V, Biggs JS, *et al*. A genomic view of trophic and metabolic diversity in clade-specific *Lamellodysidea* sponge microbiomes. *Microbiome* 2020; **8**(1): 1-17.
97. Kelly JB, Carlson DE, Low JS, Thacker RW. Novel trends of genome evolution in highly complex tropical sponge microbiomes. *Microbiome* 2022; **10**(1): 164.
98. Chaib De Mares M, Jiménez DJ, Palladino G, Gutleben J, Lebrun LA, Muller EEL, *et al*. Expressed protein profile of a Tectomicrobium and other microbial symbionts in the marine sponge *Aplysina aerophoba* as evidenced by metaproteomics. *Sci Rep* 2018; **8**: 11795.
99. Deborah Zamble by, Rowińska-Żyrek M, Kozłowski H, rodrigue agnes, albareda M, Mandrand-berthelot M, *et al*. Chapter 12 Nickel in microbial physiology-from single proteins to complex trafficking systems: nickel import/export. *Metallobiology Series*. 2017.
100. Lessard IAD, Walsh CT. VanX, a bacterial D-alanyl-D-alanine dipeptidase: Resistance, immunity, or survival function? *Proc Natl Acad Sci U S A* 1999; **96**: 11028–11032.
101. Guisbert E, Yura T, Rhodius VA, Gross CA. Convergence of Molecular, Modeling, and Systems Approaches for an Understanding of the *Escherichia coli* Heat Shock Response. *Microbiol Mol Biol R* 2008; **72**: 545–554.
102. Laport MS, Dos Santos LL, Lemos JAC, Do Carmo F. Bastos M, Burne RA, Giambiagi-Demarval M. Organization of heat shock dnaK and groE operons of the nosocomial pathogen *Enterococcus faecium*. *Res Microbiol* 2006; **157**: 162–168.
103. Camarena L, Bruno V, Euskirchen G, Poggio S, Snyder M. Molecular mechanisms of ethanol-induced pathogenesis revealed by RNA-sequencing. *PLoS Pathog* 2010; **6**: 1–14.
104. Kupper M, Gupta SK, Feldhaar H, Gross R. Versatile roles of the chaperonin GroEL in microorganism-insect interactions. *FEMS Microbiol Lett* 2014; **353**: 1–10.
105. Charles H, Heddi A, Guillaud J, Nardon C, Nardon P. A Molecular Aspect of Symbiotic Interactions between the Weevil *Sitophilus oryzae* and Its Endosymbiotic Bacteria: Over-expression of a Chaperonin. *Biochem Biophys Res Commun* 1997; **239**: 769–774.
106. Wilcox JL, Dunbar HE, Wolfinger RD, Moran NA. Consequences of reductive evolution for gene expression in an obligate endosymbiont. *Mol Microbiol* 2003; **48**: 1491–1500.
107. Stoll S, Feldhaar H, Gross R. Transcriptional profiling of the endosymbiont *Blochmannia floridanus* during different developmental stages of its holometabolous ant host. *Environ Microbiol* 2009; **11**: 877–888.
108. McCutcheon JP, McDonald BR, Moran NA. Convergent evolution of metabolic roles in bacterial co-symbionts of insects. *Proc Natl Acad Sci U S A* 2009; **106**: 15394–15399.

109. Fares MA, Ruiz-González MX, Moya A, Elena SF, Barrio E. GroEL buffers against deleterious mutations. *Nature* 2002; **417**: 398–398.
110. Login FH, Séverine Balmand, Agnès Vallier, Carole Vincent-Monégat, Aurélien Vigneron, Michèle Weiss-Gayet, *et al*. Antimicrobial peptides keep insect endosymbionts under control. *Science* 2011; **334**(6054): 362-365.
111. Fares MA, Moya A, Barrio E. GroEL and the maintenance of bacterial endosymbiosis. *Trends Genet* 2004; **20**: 413–416.
112. Moitinho-Silva L, Seridi L, Ryu T, Voolstra CR, Ravasi T, Hentschel U. Revealing microbial functional activities in the Red Sea sponge *Stylissa carteri* by metatranscriptomics. *Environ Microbiol* 2014; **16**: 3683–3698.
113. Bhavsar RB, Makley LN, Tsonis PA. The other lives of ribosomal proteins. *Hum Genomics* 2010; **327**: 4: 1-18.
114. King GM, Weber CF. Distribution, diversity and ecology of aerobic CO-oxidizing bacteria. *Nat Rev Microbiol* 2007; **5**: 107–118
115. King GM. Molecular and Culture-Based Analyses of Aerobic Carbon Monoxide Oxidizer Diversity. *Appl Environ Microbiol* 2003; **69**: 7257–7265.
116. Burke C, Steinberg P, Rusch D, Kjelleberg S, Thomas T. Bacterial community assembly based on functional genes rather than species. *Proc Natl Acad Sci U S A* 2011; **108**: 14288–14293.
117. McCutcheon JP, McDonald BR, Moran NA. Convergent evolution of metabolic roles in bacterial co-symbionts of insects. *Proc Natl Acad Sci U S A* 2009; **106**: 15394–15399.
118. Talbot JM, Bruns TD, Taylor JW, Smith DP, Branco S, Glassman SI, *et al*. Endemism and functional convergence across the North American soil mycobiome. *Proc Natl Acad Sci U S A* 2014; **111**: 6341–6346.
119. Root RB. The Niche Exploitation Pattern of the Blue-Gray Gnatcatcher. *Ecol Monogr* 1967; **37**: 317–350.
120. Blondel J. Guilds or functional groups: Does it matter? *Oikos* 2003; **100**: 223–231
121. Koch EBA, Castaño-Meneses G, Delabie JHC. The guild concept: From feudalism to community ecology. *Acta Biolo Colomb* 2019; **24**: 224–231.
122. Bauer MA, Kainz K, Carmona-Gutierrez D, Madeo F. Microbial wars: Competition in ecological niches and within the microbiome. *Microbial Cell* 2018. Shared Science Publishers OG; **5**: 215–219
